# Determination of Vibration Properties and Reliable Frequency Estimation for Synchronous Vibrations Through Improved Blade Tip Timing Techniques Without a Once-per-Revolution Sensor †

**DOI:** 10.3390/s25020489

**Published:** 2025-01-16

**Authors:** Marios Sasakaros, Luca Mann, Markus Schafferus, Manfred Wirsum

**Affiliations:** Institute of Power Plant Technology, Steam and Gas Turbines, RWTH Aachen University, 52062 Aachen, Germany

**Keywords:** blade tip timing, synchronous blade vibrations, blade fingerprints, rotor postion, NUFFT, multi-sampling, circumferential Fourier fit

## Abstract

Synchronous vibrations, which are caused by periodic excitations, can have a severe impact on the service life of impellers. Blade Tip Timing (BTT) is a promising technique for monitoring synchronous vibrations due to its non-intrusive nature and ability to monitor all blades at once. BTT generally employs a Once-per-Revolution (OPR) sensor that is mounted on the shaft for blade identification and deflection calculation. Nevertheless, OPR sensors can be unreliable, as they may be affected by shaft vibrations, and their implementation can be restricted by space constraints. Moreover, the low number of BTT sensors typically leads to under-sampled deflection signals, which consequently hinders the estimation of the vibration frequencies due to aliasing problems. For this reason, BTT is commonly accompanied by strain gauge (SG) measurements on some blades. In this paper, improved BTT techniques are presented, which enable the determination of vibration properties of synchronous vibrations without the need for an OPR sensor and ensure a reliable frequency assessment. Specifically, the blades are identified by unique characteristics resulting from manufacturing tolerances, while the blade deflections are calculated through a novel method, which relies on the impeller’s circumferential position. The proposed method enables accurate OPR-free calculation of blade deflections, by accounting for speed variations within a revolution and considering the actual blade positions on the impeller. By completely eliminating the need for an OPR sensor, the accuracy of BTT is enhanced, as the blade deflections are no longer affected by shaft vibrations, while speed variations within a revolution can be accounted for. Moreover, the implementation possibilities of BTT are improved, allowing its application in systems, where an OPR sensor cannot be instrumented due to space constraints. Subsequently, the vibration frequencies are accurately estimated, by employing an improved Multi-Sampling method based on Non-Uniform Fast Fourier Transform. This approach enables the blind analysis of BTT measurements and can identify multiple vibration frequencies. The proposed method expands the capabilities of BTT through a reliable assessment of vibration frequencies from under-sampled BTT signals. Therefore, it is no longer necessary to accompany BTT measurements with SG measurements for frequency identification. Finally, the vibration properties are determined using regression models. The proposed BTT techniques are validated through comparison with SG measurements as well as a commercial BTT system, using experimental data from a test bench of a turbocharger used for marine applications. The vibrations were recorded under real operating conditions, thus demonstrating the industrial applicability of the proposed BTT evaluation procedure.

## 1. Introduction

Synchronous blade vibrations can significantly affect the service life of turbomachine impellers, as their occurrence can lead to limit cycle oscillations that, over time, increase the risk of component failure due to high cycle fatigue. Therefore, the precise estimation of resonance operating points and quantification of vibration amplitudes are essential for an accurate assessment of impeller service life.

Synchronous blade vibrations are primarily generated by the non-uniform flow field in the circumferential direction, which exerts periodic pressure fluctuations on the blades. These vibrations are characterized by their frequencies, which correspond to integer multiples of the rotational frequency, known as Engine Orders (EOs). Wake vortices and potential excitation through the stationary guide vanes [1,2,3,4], as well as the non-uniformity of the flow field due to the geometry of the spiral housing [5,6,7], have been identified as the main excitation sources.

For the measurement of blade vibrations, two techniques are commonly employed: strain gauges (SGs) and Blade Tip Timing (BTT). SGs have been extensively used for vibration measurements [3,8,9,10,11]. SGs offer a continuous signal, enabling the precise determination of the properties of several vibrations simultaneously through traditional Short-Time Fourier Transform (STFT) approaches. However, they can only monitor individual blades, and the number of observed blades is constrained by the available telemetry channels. Moreover, they are characterized by their limited service life, as they are subjected to centrifugal loads, and their application can alter the vibration characteristics of the blades. In fact, Beirow et al. [8] showed that SGs can lead to increased natural frequencies of the instrumented blades.

In contrast, BTT uses contactless sensors, which are installed in the housing around the impeller circumference, to measure blade vibrations. BTT has been used by various studies in recent years for vibration measurements [11,12,13,14,15,16]. BTT measures the time of arrival (TOA) of the blades at the BTT sensors. The tangential deflection of the blade tips, at the axial plane of the BTT sensors, can be determined through the difference between the expected TOA of the non-vibrating blades and the measured TOA. The calculated deflections can then be used to determine the vibration characteristics. For the calculation of the expected TOA, BTT generally employs a Once-per-Revolution (OPR) sensor, which is mounted on the shaft, to deliver a reference time per revolution. This reference time is additionally used for blade identification, i.e., to identify which blade a TOA belongs to.

The primary advantages of BTT derive from its non-intrusive nature. As the BTT sensors are instrumented in the housing around the impeller, BTT does not affect the vibration properties of the blades. Additionally, the BTT sensors have significantly greater durability, since they are not applied on the blades and, thus, are not subjected to centrifugal loads. A crucial advantage of BTT lies in its ability to measure the vibration properties of all blades simultaneously. Hence, properties such as amplitude and frequency mistuning, which require the vibration properties of all blades, can be accurately estimated. This enables precise mistuning assessment and amplitude overshoot identification.

Even though BTT offers significant advantages over SG, the use of BTT can be hindered by several issues. One issue arises from the employment of the OPR sensor for the calculation of the blade deflections. In this aspect, the quality of the OPR signal can dramatically affect the accuracy of BTT. Particularly, Zhang J. et al. [17] showed that the error of BTT systems, which utilize an OPR sensor, can be considerably higher compared to systems without OPR signals. The authors mentioned that shaft bend and torsional vibrations can affect the OPR signal and thus lead to high noise components. Furthermore, the implementation of the OPR sensor can be restricted by space constraints, while a damaged OPR sensor can cause the failure of the entire BTT system. Last but not least, an OPR sensor is incapable of monitoring the speed variation within a revolution.

Another major issue associated with BTT is the limited number of sensors. Due to the low number of sensors, BTT offers a non-continuous measurement with only a few sample points per revolution. Hence, the deflection signal is typically under-sampled and does not satisfy the Nyquist criterion [18]. Thus, the deflection signal cannot be perfectly reconstructed, and multiple frequencies fit to the signal due to aliasing. These frequencies are called replications and prohibit the identification of the true vibration frequency. As a result, conventional Fourier Transform approaches do not provide meaningful results, as the true vibration frequency cannot be distinguished from the replications.

To overcome the problems correlated with the under-sampled BTT signal and determine the vibration properties of the blades, regression models based on the Least Square Method (LSQM) are commonly employed. Specifically, the Circumferential Fourier Fit (CFF) method has been extensively used by various researchers [19,20,21,22,23] in recent years. CFF tries to fit undamped sine oscillations to the BTT deflection signals. However, this method requires a prior knowledge of the possible occurring EOs.

CFF determines the phase and amplitude of each EO along with an error term, which indicates how good the EO fits to the BTT data. The user must then identify the true EO based on the CFF results. In this aspect, there is always the possibility that the true EO lies outside of the frequency interval provided by the user. Moreover, EOs, which fit to the deflection signal due to the aliasing effect, can be incorrectly identified as the true EO due to their low error term. As a result, automated identification methods are prone to errors and significant user effort is required to correctly identify the true EO. For this reason, SGs are usually instrumented alongside BTT on some blades for the identification of vibration frequencies.

This paper presents a comprehensive evaluation procedure for BTT data, starting from the determination of the TOAs from raw signals to the calculation of blade vibration properties. The proposed methods aim to address the challenges associated with BTT, namely the dependence on the OPR sensor for calculating blade deflections and the problematic estimation of the vibration frequency due to the under-sampled deflection signal. Hence, the main contributions of this paper are as follows: (1) the novel Rotor Position method for accurate OPR-free calculation of blade deflections and (2) an improved Multi-Sampling method for reliable vibration frequency assessment in under-sampled BTT signals.

The OPR signal can be omitted from the BTT evaluation procedure by identifying the blades and calculating the blade deflections using OPR-free methods. In this study, the blades are identified without an OPR signal by utilizing their distinct geometric characteristics resulting from manufacturing tolerances. Subsequently, the novel Rotor Position method is introduced for the calculation of blade deflections without an OPR sensor. This method calculates the blade deflections by subtracting the sensor positions from the blade circumferential positions. A precise estimation of the blade circumferential positions is acquired by performing a numerical integration of the rotor rotational frequency over time and considering the actual blade positions on the impeller. The rotor rotational frequency is calculated by considering all TOAs in a revolution, resulting in a high-resolution signal capable of accounting for speed variations within a revolution.

By completely eliminating the need for an OPR sensor, the accuracy and implementation possibilities of BTT are enhanced. Specifically, a more precise estimation of the blade deflections is enabled, as the blade deflections are no longer affected by shaft vibrations [17], while speed variations within a revolution can be accounted for. Moreover, the implementation possibilities of BTT are enhanced, allowing its application in systems where an OPR sensor cannot be instrumented due to space constraints.

In addition, this paper proposes an improved Multi-Sampling method [24,25,26] for the reliable frequency assessment of synchronous vibrations in under-sampled BTT signals. This method utilizes the distinctive aliasing patterns generated by different non-equidistant sensor layouts to identify the vibration frequency. Initially, the frequency spectra of different sensor layouts are calculated through Non-Uniform Fast Fourier Transform (NUFFT) analysis. Subsequently, to isolate the vibration frequency, which is common to all sensor layouts, the inter-spectrum is calculated. By employing an automated analysis of the inter-spectrum across all blades and resonance events within a given speed range, the vibration frequency is identified. This approach enables the blind analysis of BTT measurements and can identify multiple vibration frequencies. The proposed method improves the capabilities of BTT through a reliable assessment of vibration frequencies, eliminating the need for parallel strain gauge measurements for frequency identification.

This paper is structured as follows: Section 2 presents a literature review of OPR-free BTT techniques for calculating blade deflection, comparing the Rotor Position method introduced in this manuscript with earlier OPR-free methods to highlight its unique approach and advantages. For this purpose, a literature overview of frequency identification methods in under-sampled BTT signals is presented, and the improved Multi-Sampling method proposed in this paper is compared with these approaches. Section 3 provides a brief description of the test bench and the measurement systems that are employed to acquire the experimental data used for development and validation of the presented methods.

In the following Section 4, the developed BTT evaluation procedure is thoroughly described. This chapter is divided into several sections, each explaining a different task of the evaluation process. Initially, a TOA position along the voltage pulse generated by a blade passing in front of a BTT sensor is selected. Then, the blade identification procedure is introduced, and, subsequently, the calculation process of the Rotor Position method is presented. Next, the improved Multi-Sampling method, which is employed for assessing the vibration frequency, is explained. In the last section of this chapter, the CFF analysis is utilized for calculating the vibration amplitude and phase. The proposed techniques are validated through comparison with a commercial BTT system as well as SG measurements using experimental data acquired from a turbocharger test bench during real operation. Finally, Section 5 provides the conclusion and outlook of this study.

This manuscript is an extended version of the GT2024-122642 [27] conference paper published in the proceedings of the 2024: Turbomachinery Technical Conference and Exposition.

## 2. Literature Review

Before presenting the developed evaluation procedure, which aims to address two significant challenges in BTT data analysis by providing an accurate OPR-free calculation of blade deflections via the Rotor Position method, and reliably identifying vibration frequencies through an improved Multi-Sampling method, it is necessary to review the approaches introduced in earlier publications for overcoming these problems. In the following sections, a literature review of both non-OPR methods for calculating blade deflections and frequency identification methods from under-sampled BTT signals is provided, with emphasis on their assumptions and limitations. In the concluding paragraphs of these sections, the methods presented in this paper are briefly described and subsequently compared with those proposed in earlier publications to highlight their unique approach and underline their advantages.

### 2.1. Non-OPR Methods for Calculation of Blade Deflection

In recent years, several BTT methods have emerged for assessing blade deflections without relying on an OPR signal. Russhard [28] proposed the Straight Line Fitting (SLF) technique for deriving virtual OPR signals from TOA data under the assumption of constant speed within a revolution. Building upon the same assumption, Chen K. et al. [29] introduced the Compound Reference method, enhancing accuracy by utilizing the blade at the midpoint of the SLF interval as reference.

More recently, He et al. [30] and Daga et al. [31] suggested generating a virtual reference signal based on a moving average of the TOA data, resulting in a different reference time for each blade within a revolution. Although this method can capture linear speed changes within a revolution, its accuracy is limited under non-linear speed variations. In addition, Wang W. et al. [32] introduced the Coupled Vibration Analysis method, which utilizes one blade as a reference, while analyzing the remaining blades relative to it. For reliable results, this approach requires the presence of mistuned blades.

To address variable speed within a revolution, Zhang J. W. et al. [33] proposed using multiple reference times around the impeller circumference, by dividing the impeller into multiple reference phases. In this approach, the rotational speed between two reference phases is considered constant, while the influence of rotational speed variation on the measured TOAs is ignored. Fan C. et al. [34] introduced a self-correcting BTT method to determine the expected TOA. By utilizing data from a reference probe, speed changes are assessed, and fitting coefficients are corrected accordingly, enabling precise derivation of the expected TOA. Although this method is capable of capturing non-linear speed variations, it does not consider the impact of blade vibrations on the reference probe, which can influence the estimated speed change.

Recently, Fan Z. et al. [35] presented an improved Multiple-per-Revolution (MPR) method, which generates MPR signals by fitting the TOA data via the least-squares polynomial method. In this approach, the MPR signals, which serve as theoretical TOAs of non-vibrating blades, are derived under the assumption of a linear speed change within a revolution and consideration of the actual blade positions on the impeller. Wang W. et al. [36] addressed challenges during rapid speed changes using Savitzky–Golay filters (SGFs) for noise reduction and calculating the speed of each blade separately at each sensor. By establishing a model correlating the expected TOA with actual blade position, the blade deflection is calculated. Even though this method can improve the accuracy of BTT under speed fluctuations, the use of SGF can introduce false blade deflections due to fitting errors.

Lastly, Guo et al., Zhang J. et al., and Wang Z. et al. [17,37,38] proposed an alternative approach that utilizes deflection differences between two BTT sensors to identify vibration parameters without an OPR sensor through regression models. However, since this method does not provide blade deflections, it is not compatible with signal processing techniques used for vibration frequency identification and signal reconstruction.

In summary, the most recent non-OPR methods aim to generate accurate theoretical TOAs of non-vibrating blades by considering speed variation within a revolution and actual blade positions through high-order fitting of the measured TOAs. Blade deflections are then calculated by comparing these theoretical TOAs with the measured TOAs. In contrast, the Rotor Position method proposed in this paper calculates blade deflections in the angle domain by comparing blade and sensor positions. First, a rotational frequency is calculated for each BTT sensor to account for speed variation within a revolution. To minimize the influence of blade vibrations on the rotational frequency, the rotational frequency of the rotor is obtained by averaging the rotational frequencies of the BTT sensors. The rotor circumferential position is then determined through integration of the rotational frequency over time. Subsequently, the blade circumferential positions are calculated by interpolating the rotor circumferential position to the measured TOAs and considering the actual blade positions on the impeller. Blade deflections are finally determined by subtracting sensor positions from blade positions. By employing this approach, an estimation of the expected TOAs through high-order polynomial fit is avoided, while speed fluctuations and actual blade positions are accounted for. In addition, the impact of occurring vibrations on the rotational speed is minimized.

### 2.2. Frequency Identification Methods

In recent years, numerous methods have been proposed for frequency identification in under-sampled BTT signals. Among these, compressive sensing (CS) has been extensively employed. CS exploits the signal sparsity in certain domains for signal reconstruction from significantly fewer samples. Lin et al. [39] pioneered the application of CS techniques in BTT by introducing sparse reconstruction models for vibration frequency identification. Pan et al. [40,41] expanded upon the work of Lin et al. [39] to enhance noise robustness and enable feature recognition under nonlinearity. Chen et al. [42] introduced a CS model for spectrum reconstruction under variable speed conditions. Additionally, Bouchain et al. [43] and Xu et al. [44] proposed CS algorithms for accurate amplitude estimation. Despite these improvements, the effectiveness of CS methods in reconstructing spectra containing synchronous vibrations remained limited. To address this issue Dong et al. [45,46] recently introduced the subspace pursuit algorithm. This recovery algorithm overcomes challenges related to selecting valid atoms by allowing multiple atoms to be chosen simultaneously.

Nevertheless, CS methods still encounter several challenges. One significant issue is the sensitivity of CS methods to sensor layout [39,40,42,45,47,48,49]. Suboptimal sensor placements can lead to inaccurate reconstructions and parameter estimations. Additionally, CS methods demand prior knowledge of the number of frequency components [50] and involve computationally intensive iterative processes. Furthermore, accurate amplitude estimation for synchronous vibrations remains an area of concern.

Cao et al. [50] focused on the recovery of covariance information from BTT signals, presenting coprime sampling-based BTT and nested sampling-based BTT methods for spectrum reconstruction and parameter identification. Unlike CS methods that directly reconstruct signal waveforms, compressed covariance sensing methods focus on reconstructing covariance information, thus leading to significantly improved computational efficiency. However, the maximum recoverable frequency is dependent on the number of coprime and nested sampling intervals, which is determined by the sensor layout. In addition, Cao et al. [51] introduced a time delay-based spectrum reconstruction method for BTT signals, applicable for both synchronous and asynchronous vibrations. In this method, the identification of synchronous vibrations is based on comparing the aliasing spectra of each BTT sensor with of all possible frequency combinations and finding the frequency combination with the best fit. For successful spectrum reconstruction, certain requirements relating to the probe number and layout must be satisfied.

Another widely employed approach for frequency identification in BTT is Multiple Signal Classification (MUSIC). Leveraging the orthogonality of signal and noise subspace, MUSIC detects signal arrival direction through spectral peak search. Wang Z. et al. [52] and Liu et al. [53] improved MUSIC for BTT signal reconstruction aiming at enhanced frequency identification. Yet, challenges including frequency aliasing, restrictions in the number of identified frequencies, and amplitude information loss persist. More importantly, the effectiveness of MUSIC in detecting synchronous vibrations remains limited [49,50,52,53,54,55,56].

This paper introduces an improved Multi-Sampling method [24,25,26] for automatically identifying frequencies of synchronous vibrations in BTT signals. This technique leverages the distinctive aliasing patterns generated by different non-equidistant sensor layouts. Each sensor layouts produces unique replication patterns, with the true vibration frequency present across all layouts. To isolate the vibration frequency, which is common to all sensor layouts, the inter-spectrum is calculated. To enable reliable frequency identification, this method incorporates an automated analysis of the inter-spectrum across all blades and resonance events within a given speed range. Instead of engaging in signal reconstruction, the estimated frequencies serve as input for CFF, which efficiently compute vibration amplitude and phase.

The implementation of the Multi-Sampling method enables blind analysis, eliminating the need for frequency estimation and facilitating automated frequency identification. Unlike iterative complex signal reconstruction techniques, the proposed method operates non-iteratively, reducing computational overhead. Moreover, this method demonstrates robustness across variable speed conditions and does not require strict sensor layouts.

## 3. Experimental Data Acquisition

**Figure 1:**
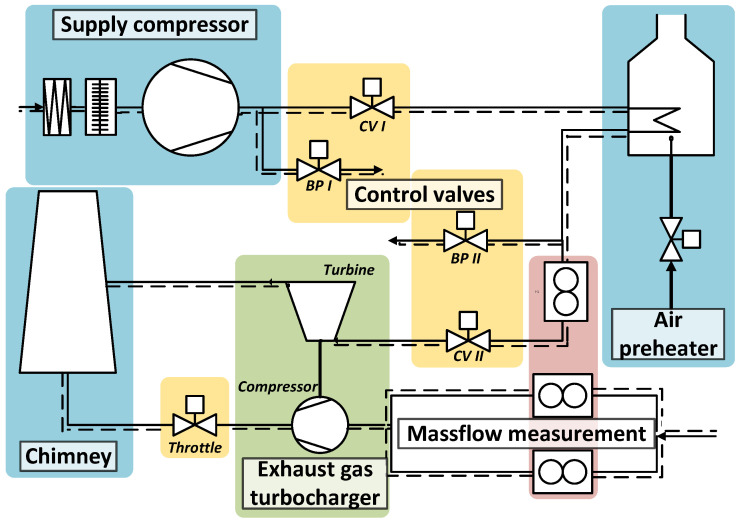
Illustration of the test bench setup.

The experimental data used in this study for the development and validation of the proposed BTT evaluation procedure were acquired from a turbocharger test bench located at the Institute for Power Plant Technology, Steam and Gas Turbines of RWTH Aachen University [57]. In this test bench, a six-stage radial compressor provided air to the turbocharger turbine. To simulate the actual operating conditions of the radial turbine, the pressure of the supplied air was adjusted through a series of valves, while its temperature was regulated using a natural gas-fired heat exchanger. The turbocharger compressor drew air at atmospheric conditions, and its outlet pressure was set via a throttle valve. This configuration allowed the precise adjustment of the investigated operating point, enabling vibration measurements under real operating conditions. The test bench is illustrated in Figure 1. A detailed description can be found in previous publications [11,57].

The exhaust gas turbocharger employed in the experiments was provided by Kompressorenbau Bannewitz GmbH (Bannewitz, Germany) (KBB) and is used for marine applications. To avoid exposing the rotor to high stresses for long time periods, the tests were performed by sweeping through the resonance. This was achieved by gradually increasing or decreasing the rotational speed.

**Figure 2:**
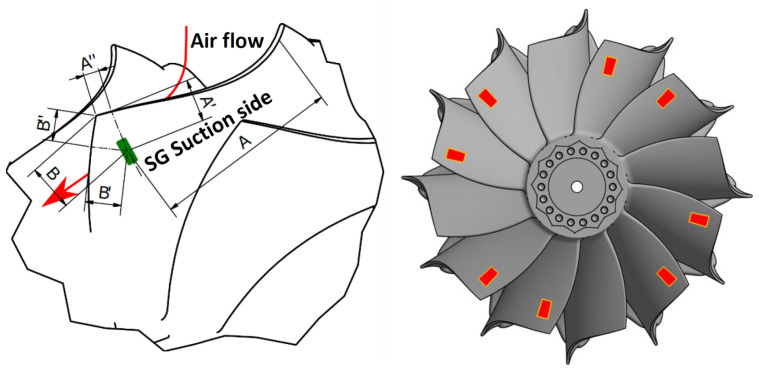
Position and orientation of SGs on blades (**left**) and SG distribution on the impeller (**right**).

Blade vibrations were measured using two different techniques. SGs were attached to eight blades, while eight optical BTT sensors were distributed non-equidistantly around the impeller circumference to measure the TOAs. To determine the optimal position and orientation of the SGs on the blade surfaces, as well as the axial plane of the BTT sensors, the vibration modes of the impeller were examined prior to the experiments through numerical modal analysis utilizing FEM models. The position and orientation of SGs on the blades as well as their distribution on the impeller are shown in Figure 2. Since most of the impeller modes exhibit the largest tangential deflections at the blade tip of the trailing edge, the axial BTT plane was positioned 2 mm upstream of the blade trailing edge.

During operation, the laser beams were generated from a laser module containing laser diodes that emit light at a wavelength of 450 nm. The intensity of each laser diode could be adjusted individually. The sensors directed the laser beams onto the blade tips, where the light was reflected and guided back to the detector module through fiber-optic probes. At the detector module, a photodiode converted the optical signal into an analog voltage signal, which was then amplified. The laser module and the detector module are part of a laser box, which is an in-house construction.

**Figure 3:**
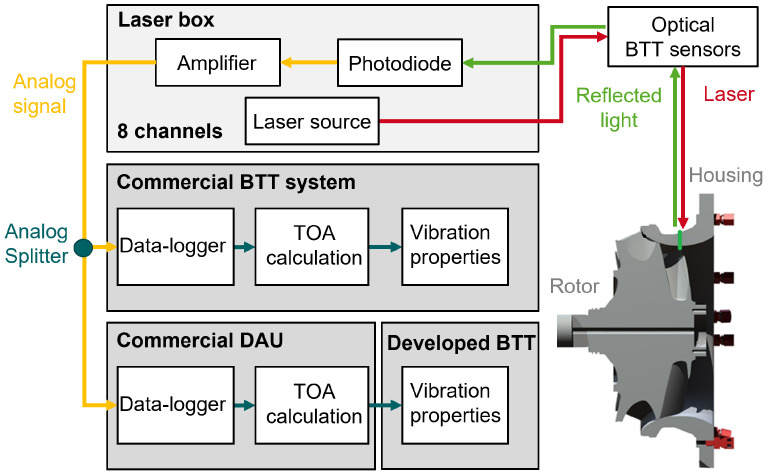
Configuration of the BTT measurement systems.

The raw analog signals of the BTT sensors were split and provided to both a commercial BTT system manufactured by Hood Technology and a commercial Data Acquisition Unit (DAU) from Prime Photonics. The TOAs used for the evaluation procedure proposed in this paper were acquired from the DAU of Prime Photonics, while the vibration properties determined by the SGs and the commercial BTT system were utilized for the comparison and validation of the presented methods. An illustration of the BTT measurement configuration is provided in Figure 3. A detailed description of the test bench and a comprehensive analysis of the vibration measurements can be found in previous publications [11,57].

## 4. Blade Tip Timing Processing Sequence

**Figure 4:**
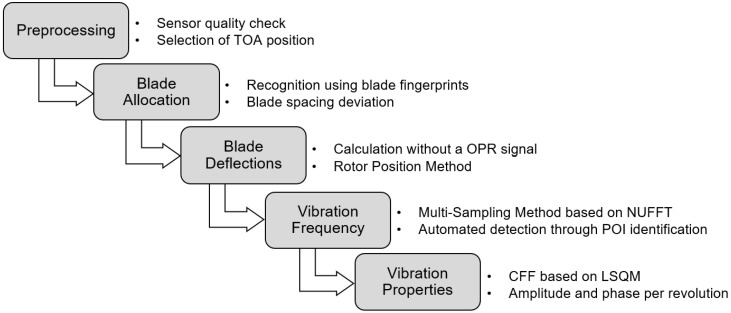
Workflow chart of Blade Tip Timing processing sequence.

The proposed BTT processing sequence is divided in smaller blocks, which each performs a certain evaluation task. A workflow chart of the BTT evaluation procedure is presented in Figure 4. Initially the TOAs are formatted in a preprocessing block. After the preprocessing, blade identification must be performed. In this block, the TOAs are allocated among the impeller blades. Next, the blade deflections are calculated through the novel Rotor Position method, which will be thoroughly described in a following section. Both the blade allocation and the deflection calculation do not rely on an OPR signal. Afterwards, the vibration frequency can be reliably determined through the improved Multi-Sampling method, which is based on NUFFT approaches. Even though the proposed method can deliver a reliable assessment of the vibration frequency, an accurate estimation of the vibration properties is not possible. Therefore, it is necessary to employ other methods for the determination of the vibration amplitude and phase. For this reason, the vibration frequency is provided to a conventional CFF analysis, where the amplitude and phase of each blade are determined for each revolution. By estimating the vibration frequency, a user-defined frequency interval is no longer required, and the number of EOs that are evaluated by the CFF method is significantly reduced. As a result, an automated evaluation procedure is enabled, while parallel strain gauge measurements for frequency identification become obsolete.

### 4.1. Data Acquisition and Preprocessing

**Figure 5:**
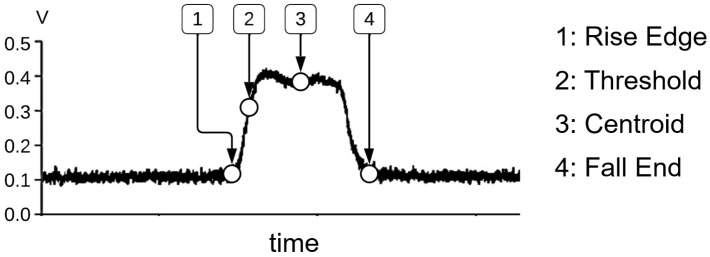
TOA positions along a voltage pulse generated from a blade passing in front of a sensor.

The evaluation process begins with the acquisition of the TOAs of the blades. The TOAs are obtained using a commercial DAU from Prime Photonics. This DAU digitally calculates the TOAs, allowing determination at several positions along the voltage pulse generated when a blade passes in front of a BTT sensor. An exemplary voltage pulse is depicted in Figure 5. As shown, TOAs are determined for the following points: Rise Edge, Threshold, Centroid, and Fall End. The Rise Edge TOA corresponds to the beginning of the rising edge of the voltage pulse. The Threshold TOA is located along the rising edge, at a voltage value automatically adjusted by the system, e.g., at 80% of the peak voltage. The Fall End TOA is positioned at the end of the falling edge of the signal. The Centroid TOA lies at the center of the voltage pulse and is defined by the mean time of all the points on the voltage pulse. From these positions, a specific TOA position must be selected for further evaluation.

TOAs can be influenced by several factors related to the experimental setup and sensor characteristics. Specifically, TOA fluctuations may result from the defused reflection of the laser light of the optical sensors on the blade tips, as well as from oscillations in the sensor positions within the housing. Additionally, the condition of the fiber-optic sensors can significantly impact the measured TOAs. Micro-cracks in the fibers due to bending, contamination from dust or moisture particles, and material degradation can profoundly affect signal quality.

**Figure 6:**
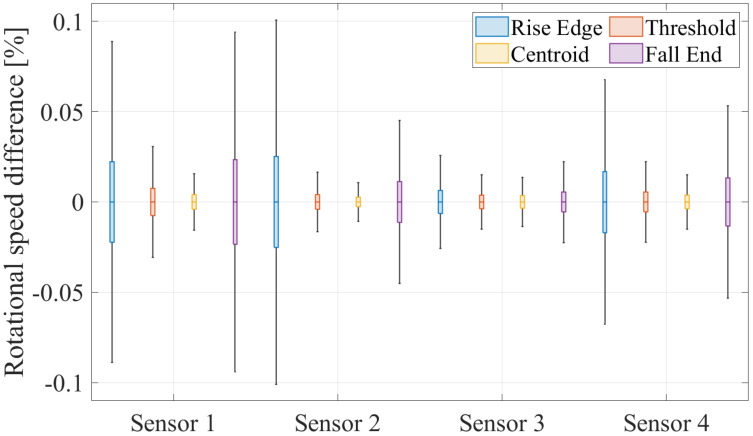
Boxplot diagram of the difference between the rotational speed determined by a TOA position and the average speed over all TOA positions for four exemplary sensors in a non-vibration speed range of approximately 30,700 RPM.

All these factors influence the rotor speed calculated from the TOA positions. Therefore, assessing the stability of a TOA position, i.e., the extent to which it is affected by these factors, can be effectively achieved by examining the rotor speed. To select a reliable TOA position, the stability of each TOA position is evaluated by analyzing the rotor speed within a speed range where no vibrations occur. For each TOA position, the rotor speed is calculated and subsequently compared to the average speed across all TOA positions. Figure 6 presents a boxplot diagram, which illustrates the differences between the speeds calculated from the individual TOA positions and the average speed for four exemplary sensors, at a rotational speed of approximately 30,700 RPM. This dataset contains TOA from more than 40,000 revolutions.

The fluctuation range is utilized to assess the TOA positions. The deviation from the average speed depends significantly on the sensor, highlighting the impact of sensor condition on signal quality. The Rise Edge and Fall End TOA positions appear particularly sensitive to sensor condition, as significant differences in speed deviations are observed between individual sensors for these positions. In contrast, the Centroid and Threshold TOA positions show only minor differences in speed deviation between sensors, indicating their enhanced stability. The stability trend of the TOA positions is consistent across all sensors. Both the Centroid and Threshold TOA positions exhibit significantly lower variations compared to the Rise Edge and Fall End positions. Therefore, they can both be used for further evaluation.

To ensure that TOA data, which are forwarded to subsequent evaluation blocks, is of good quality, the preprocessing block determines an appropriate TOA position. Furthermore, TOA data from BTT sensors with poor signal quality are omitted. The quality of the sensors is assessed similarly to the stability evaluation of the TOA positions. Specifically, BTT sensors exhibiting significantly higher speed fluctuations compared to other sensors are excluded from further evaluation. Finally, the remaining data are synchronized, as during data acquisition, the BTT sensors do not begin recording simultaneously.

### 4.2. Blade Identification

To assign the TOAs to the blades, the blade “fingerprints” are utilized. The blade “fingerprints” are distinct geometric characteristics of the blades, which are derived by the tolerances of the impeller’s manufacturing method. In this aspect, features such as the blade thickness or the blade position on the impeller are subject to minor variations, which lie in the manufacturing tolerance range. Hence, they can be used for blade identification. Aligned with numerous OPR-free BTT approaches [28,29,31,34,35,36,37], the method presented in this paper uses the circumferential angle between the blades for blade identification.

The circumferential angle between the blades can be calculated by comparing the time difference between the TOA of two consecutive blades at the same BTT sensor with the impeller’s rotation period. To remove errors due to noise, the circumferential angle is averaged over multiple revolutions as demonstrated in Equation (1). Here, *s* indicates the observed sensor, *i* the observed revolution, and *b* the observed blade.

(1) $$\begin{array}{c} \phi_{s,b\to b+1}=\frac{1}{I}\sum_{i=1}^{I}2\pi \frac{TOA_{s,i,b+1}-TOA_{s,i,b}}{TOA_{s,i+1,b}-TOA_{s,i,b}}\\ TOA_{s,i+1,b}=TOA_{s,i,b+B}\\ s=1,2,...,S\;,\;i=1,2,...,I\;,\;b=1,2,...B \end{array}$$

**Figure 7:**
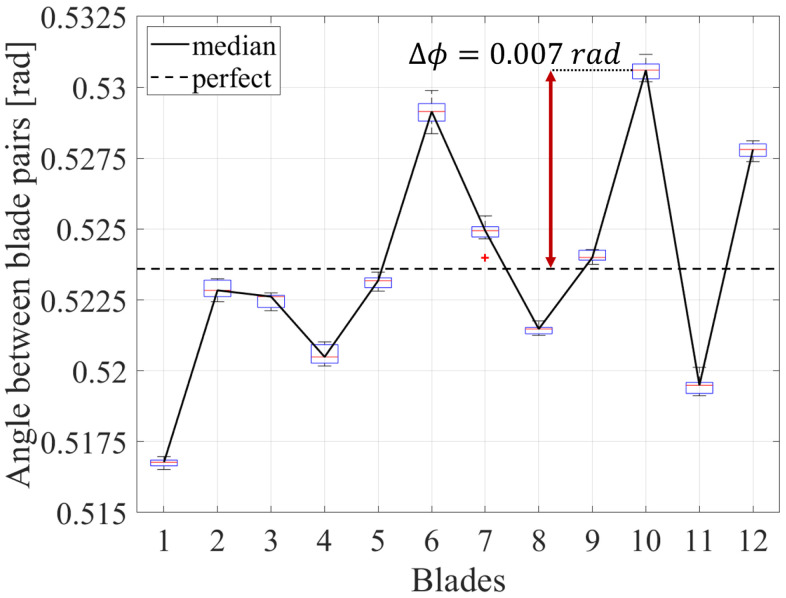
Angle between the blade pairs of a cast-manufactured impeller.

**Figure 8:**
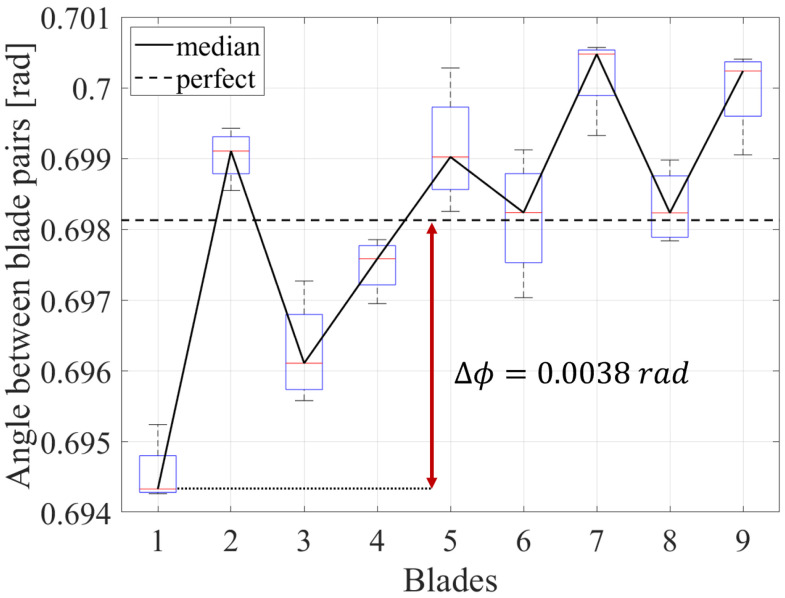
Angle between the blade pairs of a milled impeller.

Figure 7 and Figure 8 illustrate the angle between the blade pairs for two different impellers. The black line indicates the median angle between the blade pairs over all BT sensors, while the boxes illustrate the variation range between the sensors. In addition, the black dashed line indicates the angle for a perfectly manufactured impeller, which has no blade spacing variations. A clear difference between the angle of adjacent blades can be observed, which leads to a distinctive fingerprint pattern of the impeller. As this pattern derives from the manufacturing tolerances, it is unique to each impeller. This fingerprint pattern can be used for blade identification. In this case, the blade with the smallest distance to the neighboring blade is defined as the first blade.

The method was successfully applied on two impellers, which were manufactured with different processes. The first impeller was cast-manufactured, while the second impeller was milled from a solid block. As shown in Figure 7 and Figure 8, the blade “fingerprints” rely heavily on the manufacturing method. In fact, the variation range of the angle between blade pairs of the cast-manufactured impeller is approximately two times higher than the one observed at the milled impeller. This can be explained by the lower tolerances of the mill manufacturing process. Nevertheless, the method can consistently identify the blades regardless of the manufacturing process.

### 4.3. Calculation of Blade Deflections

After the blade identification and the allocation of the TOAs to the blades, the deflections of the blade tips can be calculated. For this task, the novel Rotor Position method is employed, which does not rely on an OPR signal. Unlike other OPR-free methods that perform polynomial fit of the measured TOAs to generate reference times, which are then subtracted from the measured TOAs for the calculation of blade deflections, the Rotor Position method calculates blade deflections by subtracting the sensor positions from the blade circumferential positions. The blade circumferential positions are obtained by integrating the rotor rotational frequency over time while accounting for the actual blade positions on the impeller. By considering all TOAs within a revolution, a highly sampled rotational frequency is derived that captures speed variations occurring within a revolution.

Initially, the rotational frequency $f_{s}$ of the rotor at each BTT sensor is calculated through the time difference of two consecutive TOAs of a blade as shown in Equation (2). This is performed for all blades, so that the query points of the rotational frequency in a revolution equals the number of blades.

(2) $$f_{s,i}=\left(\begin{array}{c} f_{s,i,1}\\ \vdots \\ f_{s,i,B} \end{array}\right)\;,\;f_{s,i,b}=\frac{1}{TOA_{s,i+1,b}-TOA_{s,i,b}}$$

Moreover, a rotor time $t_{R}$ is determined by the mean time of two consecutive TOAs of a blade at a BTT sensor (Equation (3)), which corresponds to the rotational frequency calculated by the same TOAs. Thus, the query points of the rotor time in a revolution equals the number of blades multiplied by the number of sensors.

(3) $$t_{R,s,i}=\left(\begin{array}{c} t_{R,s,i,1}\\ \vdots \\ t_{R,s,i,B} \end{array}\right)\;,\;t_{R,s,i,b}=\frac{TOA_{s,i,b}+TOA_{s,i+1,b}}{2}$$

Next, the rotor times of multiple revolutions are sorted in ascending order, according to Equation (4). Here, *q* indicates a query point of data sorted in ascending order:

(4) $$\begin{array}{c} t_{R}=\left(\begin{array}{c} t_{R,1}\\ \vdots \\ t_{R,q}\\ \vdots \\ t_{R,Q} \end{array}\right)\;,\;t_{R,1}<\cdots <t_{R,q}<\cdots <t_{R,Q}\\ q=1,2,...,Q\;,\;Q=S*I*B \end{array}$$

To determine the rotational frequency of the rotor $f_{R}$ from the rotational frequencies measured at the BTT sensors $f_{s}$, we estimate the rotational frequency at each sensor for all rotor times. This estimation involves linear interpolation of the frequency data from each BTT sensor to match the query points of the sorted rotor time. In this process, the frequency $f_{s,q}$ corresponding to time $t_{R,q}$ which lies within the interval $\left[t_{R,s,i,b},t_{R,s,i,b+1}\right]$ is calculated according to Equation (5). This approach is founded on the assumption that the rotational frequency at each sensor changes linearly between the intervals formed by the TOAs measured at this sensor.

(5) $$f_{s,q}=f_{R,s,i,b}+\frac{t_{R,q}-t_{R,s,i,b}}{t_{R,s,i,b+1}-t_{R,s,i,b}}\left(f_{R,s,i,b+1}-f_{R,s,i,b}\right)$$

Then, the rotational frequency of the rotor $f_{R}$ is obtained by averaging the rotational frequency at the BTT sensors (Equation (6)). Through this procedure, the influence of occurring blade vibrations on the rotational frequency is minimized.

(6) $$f_{R}=\left(\begin{array}{c} f_{R,1}\\ \vdots \\ f_{R,q}\\ \vdots \\ f_{R,Q} \end{array}\right)\;,\;f_{R,q}=\frac{1}{S}\sum_{s=1}^{S}f_{s,q}$$

Subsequently, the rotor circumferential position is calculated by means of numerical integration as shown in Equation (7).

(7) $$\phi_{R}=\int_{t_{R,1}}^{t_{R,Q}}2\pi *f_{R}\;dt$$

Next, the rotor position is interpolated at the TOAs of the individual blades. In this procedure, the rotor position $\phi_{R}\left(TOA_{s,i,b}\right)$ corresponding to measured $TOA_{s,i,b}$, which lies within the interval $\left[t_{R,q},t_{R,q+1}\right]$, is calculated according to Equation (8). Here, it is assumed that a linear change in the rotor position occurs between its query points.

(8) $$\phi_{R}\left(TOA_{s,i,b}\right)=\phi_{R,q}+\frac{TOA_{s,i,b}-t_{R,q}}{t_{R,q+1}-t_{R,q}}\left(\phi_{R,q+1}-\phi_{R,q}\right)$$

Since the TOAs are influenced by the blade deflections, the calculated rotor position contains the deflection information. If a blade experiences a positive deflection, defined by the direction of rotation, the calculated rotor position is slightly smaller than the actual value. Accordingly, a negative deflection leads to a slightly larger rotor position value. The positions of the blades are calculated by adding the angle between the first blade and the observed blade $\phi_{1\to b}$ to the rotor circumferential position, as shown in Equation (9):

(9) $$\phi_{s,i,b}=\phi_{R}\left(TOA_{s,i,b}\right)+\phi_{1\to b}$$

The angle between the first blade and the observed blade $\phi_{1\to b}$ is calculated using Equation (10), which leverages the angles between the blades derived from Equation (1). In Equation (10), *c* denotes the blades located between the first blade and the observed blade. The angles between the blades are averaged over all BTT sensors, based on the assumption that these averaged values accurately represent the true values.

(10) $$\phi_{1\to b}=\sum_{c=1}^{b-1}\frac{1}{S}\sum_{s=1}^{S}\phi_{s,c\to c+1}$$

Before the calculation of the blade deflections, it is essential to calculate the sensor circumferential positions, as their actual values deviate from the targeted value due to the manufacturing tolerances of the housing. The sensor positions are calculated from the blade positions. Specifically, the angle between the reference sensor, i.e., the first sensor to record a TOA in a revolution, and every other sensor is calculated by the circumferential position difference of a blade at these sensors. This is performed for all blades and over multiple revolutions. The sensor positions are derived by averaging the angle between the sensors over all blades and revolutions as shown in Equation (11). To avoid influence from occurring vibrations, speed regions where no vibrations arise, e.g., at the beginning or end of the recording, can be used for the calculation of the sensor positions. Hence, it can be assumed that the calculated sensor positions correspond to the true sensor positions.

(11) $$\theta_{s}=\frac{1}{I}\sum_{i=1}^{I}\frac{1}{B}\sum_{b=1}^{B}\left(\phi_{s,i,b}-\phi_{1,i,b}\right)$$

The blade deflections can be determined by subtracting the sensor positions from the blade positions (Equation (12)). For every completed revolution, 2π is added to the sensor positions. Here, the radius of the impeller at the axial plane of the sensors is used.

(12) $$d_{s,i,b}=\left(\phi_{s,i,b}-\left(\theta_{s}+2\pi \left(i-1\right)\right)\right)\cdot r$$

The calculated blade deflections contain a static component and a dynamic component. The static component is caused by factors such as the centrifugal load, blade twist, etc., while the dynamic component is caused by the occurring vibrations. To correctly determine the vibration properties, it is crucial to remove the static component. The static component is removed based on the assumption of a linear relationship with the rotational speed. Initially, the static component is calculated at the beginning and end of the recording. Next, a linear relation is derived between the static component and the rotational speed under the assumption that no vibrations occur at the speed ramp edges. The static component of a blade deflection is determined by the prevailing rotational speed and is calculated through linear interpolation as outlined in Equation (13). Subsequently, the dynamic deflections are determined by subtracting the static component from the blade deflections (Equation (14)).

(13) $$\begin{array}{c} d_{st,s,i,b}=d_{st,s,1,b}+\frac{f_{R,i}-f_{R,1}}{f_{R,I}-f_{R,1}}\left(d_{st,s,I,b}-d_{st,s,1,b}\right) \end{array}$$

(14) $$\begin{array}{c} d_{d,s,i,b}=d_{s,i,b}-d_{st,s,i,b} \end{array}$$

**Figure 9:**
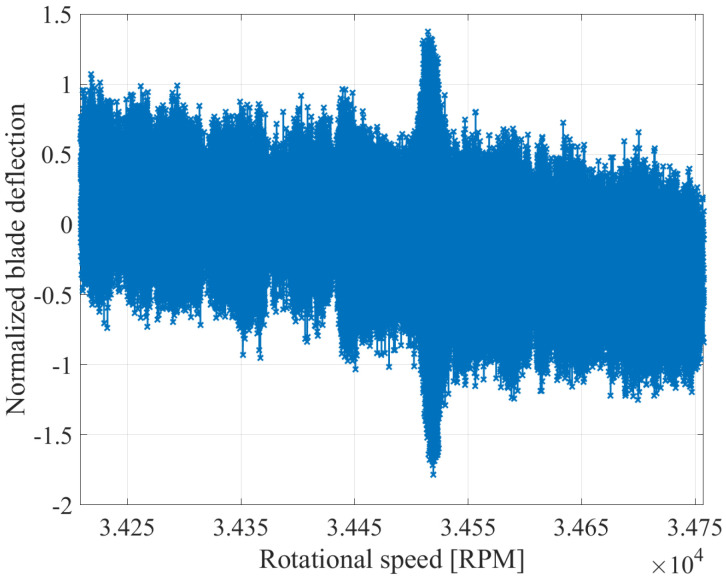
Calculated deflection with static component from all BTT sensors for an exemplary blade.

**Figure 10:**
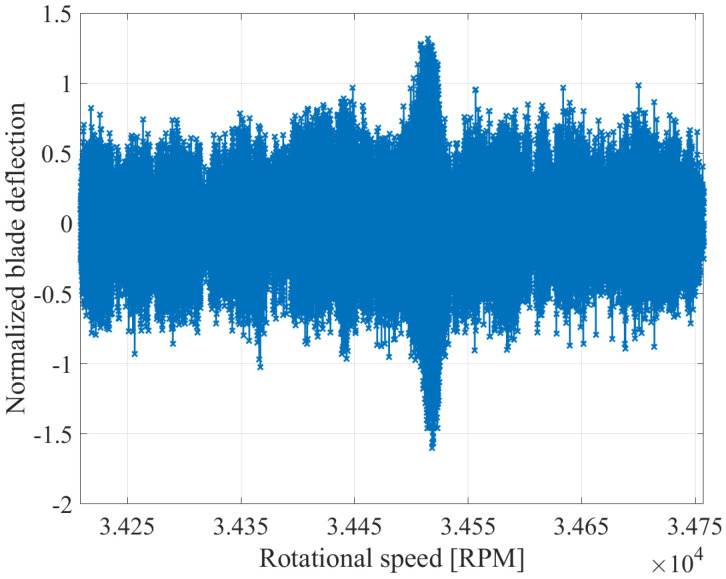
Calculated deflection without static component from all BTT sensors for an exemplary blade.

The removal of the static component is illustrated in Figure 9 and Figure 10 for an exemplary speed range, in which a vibration occurs. The figures display the deflections of a single blade at all sensors. The blade deflections, which contain the static component, are presented in Figure 9, while Figure 10 depicts only the dynamic deflections. Before the removal of the static component, the deflections oscillate around a positive value at the beginning of the recording. As the speed increases, the static component of the blades decreases. As a result, the deflections oscillate around zero at the middle of the recording, while at the end of the recording, they oscillate around a negative value. After the removal of the static component, the dynamic deflections fluctuate around zero regardless of the speed. Therefore, the hypothesis that the static component is proportional to the rotational speed is validated.

The dynamic deflections are affected by significant noise components. Minimal fluctuations of the TOAs are generated by the defused reflection of the laser light of the optical BTT sensors on the blade tips as well as by minor oscillations of the sensor positions in the housing. Moreover, small changes in the impeller axial position during operation and occurring shaft vibrations can be seen as induced noise to the calculated blade deflections. Furthermore, the condition of the fiber-optic sensors can significantly impact the measured TOAs. Micro-cracks in the fibers due to bending, contamination from dust or moisture particles, and material degradation can profoundly affect signal quality. Thus, it is essential to reduce the noise for an accurate estimation of the vibration properties.

For this task, smoothing functions are applied on the calculated blade deflections. The smoothing functions are applied for each blade and sensor pair, so that a time-synchronous averaging procedure is performed as shown in Equation (15). Here, it should be noted that the smoothing window *L*, i.e., the number of revolutions that are considered for the noise removal, is crucial for the accurate estimation of the vibration magnitudes. A small value can result in insufficient noise removal, while a large value can cause the “smoothing” of the blade deflections, leading to suppression of the occurring vibrations.

(15) $$d_{d,sm,s,i,b}=\frac{1}{L}\sum_{l=i-\frac{L}{2}}^{i+\frac{L}{2}}d_{d,s,i,b}$$

**Figure 11:**
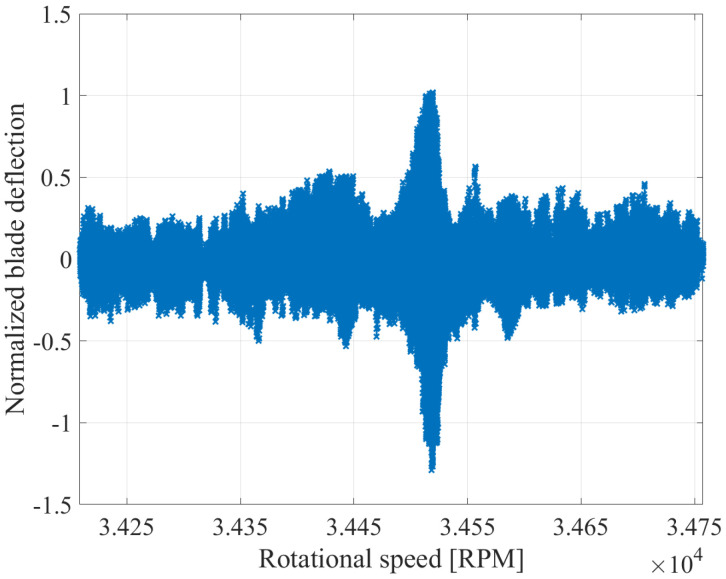
Calculated deflection with reduced noise from all BTT sensors for an exemplary blade.

To illustrate the effect of smoothing, Figure 11 depicts the blade deflections after noise reduction. The window size of the smoothing was set to 10 revolutions. Before the noise reduction (Figure 10), significant deflection fluctuations are observed. After the implementation of smoothing, a major noise reduction is achieved. As a result, a better signal-to-noise ratio is obtained, which allows easier identification of areas where blade vibrations occur.

Since the magnitude of synchronous vibrations correlates with the excitation frequency, which is defined by the rotational frequency, the impact of the window size on blade deflections is related to the change in rotational frequency during this window and, consequently, the gradient of the rotational speed. In practice, the vibration magnitude should remain constant, if the rotational frequency does not change. To investigate the influence of the chosen window size on blade deflections, the maximum rotational frequency change occurring within this window size is examined. First, the rotational frequency is segmented into sections containing a number of revolutions equal to the window size. Then, the maximum and minimum rotational frequencies in these sections are identified, and the rotational frequency change is calculated by the difference between these values. The excitation frequency change can be calculated by multiplying the rotational frequency change with the occurring EO.

**Figure 12:**
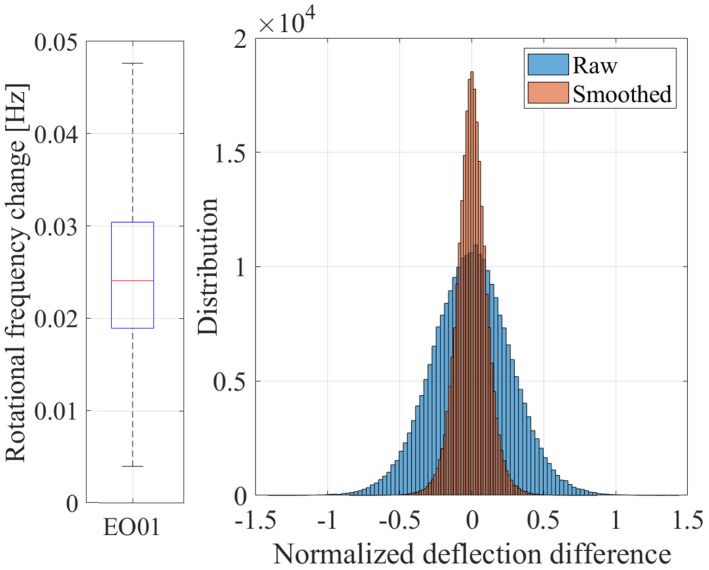
Rotational frequency change during 10 revolutions (left) and distribution of the difference between two consecutive deflections at a BTT sensor for raw and smoothed data.

The boxplot diagram in Figure 12 presents the rotational frequency change during 10 revolutions for the speed region of the blade deflections presented in Figure 11. A minor frequency change of less than 0.05 Hz is observed, translating to a relative deviation of less than 0.009%. Given that the relation between the excitation frequency change and the rotational frequency change is identical to the relation between the excitation frequency and the rotational frequency, the relative excitation frequency deviation of an EO is the same as the relative rotational frequency deviation. Therefore, the impact of the chosen window size on blade deflections should be negligible.

The noise reduction can be also illustrated by considering the difference between two consecutive dynamic deflections of a blade at a sensor. In the case of ideal measurement data, without considerable vibrations, the difference between two consecutive deflections should be close to zero. In addition, when synchronous vibrations occur, the difference between two consecutive dynamic deflections at a sensor should be minimal, assuming a minor rotational speed change and thus a small vibration magnitude difference. Hence, the difference between two consecutive dynamic deflections at a sensor can be used for noise assessment. To compare the noise between the raw and smoothed dynamic deflection, the distribution of the difference between two consecutive deflections at the same sensor for both datasets is presented in a histogram (Figure 12). Through the implemented smoothing, a substantial reduction in the deflection difference can be observed.

### 4.4. Assessment of Vibration Frequencies

After calculating the blade deflections, the vibration properties can be determined. Since the sensors are not equidistantly distributed around the impeller, the deflection signal cannot be analyzed with a Fast Fourier Transform (FFT) analysis. Instead, an NUFFT analysis must be used. NUFFT algorithms usually converts the non-uniform deflection sample points to uniformly distributed sample points, before the employment of conventional FFT approaches. For the conversion, different approaches can be used [58]. The most common methods are based on interpolation procedures [59,60,61,62,63,64,65,66]. In the proposed evaluation, the default MATLAB NUFFT algorithm is used, which employ interpolation schemes based on Gaussian bells [66,67]. The spectrum P(f) obtained from the NUFFT analysis of non-uniformly distributed sample points *p* and equidistant frequencies *f* can be expressed by Equation (16).

(16) $$P\left(f\right)=\frac{1}{N^{2}}{|\sum_{n=1}^{N}x_{n}e^{-2\pi ifp_{n}}|}^{2}$$

With NUFFT, the non-equidistant deflection signal of the blades can be analyzed. Nevertheless, due to the under-sampled nature of the BTT data, replications appear, which can be attributed to the aliasing effect. To overcome the aliasing problem, and determine the true vibration frequency, the Multi-Sampling method is used [24,25,26]. This method is based on the assumption that a specific non-equidistant sensor layout leads to a distinctive aliasing pattern. As a result, different sensor layouts produce different replication patterns, while the true vibration frequency is included in every sensor layout. Therefore, by comparing differently sampled data, the replications can be eliminated.

To identify the frequency components, which are apparent to every sensor configuration, the inter-spectrum of different sensor configurations is calculated. Hence, the replications are removed, and the remaining frequency components correspond to the true vibration frequencies. To generate datasets with different replication patterns, subgroups of BTT sensors are created, which contain different sensor configurations. The spectrum of each subgroup is calculated through an NUFFT analysis. Subsequently, the inter-spectrum is calculated according to Equation (17), where $P_{k}$ is the spectrum of a subgroup.

(17) $$P_{IS}\left(f\right)=\prod_{k=1}^{K}P_{k}\;,\;k=1,2,...,K$$

**Figure 13:**
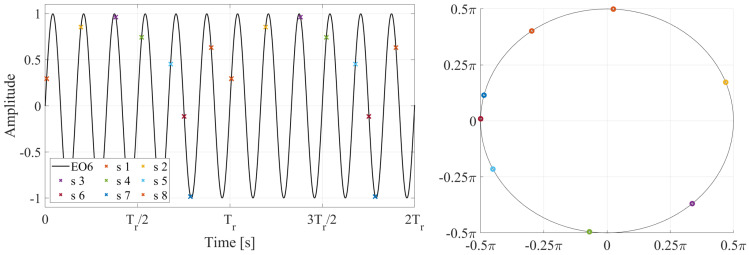
Simulated undamped oscillation of EO06, i.e., 30 Hz, over two impeller revolutions (**left**) and sensor distribution over the impeller circumference (**right**). The deflections measured by these sensors are represented on the oscillation by colored crosses.

**Figure 14:**
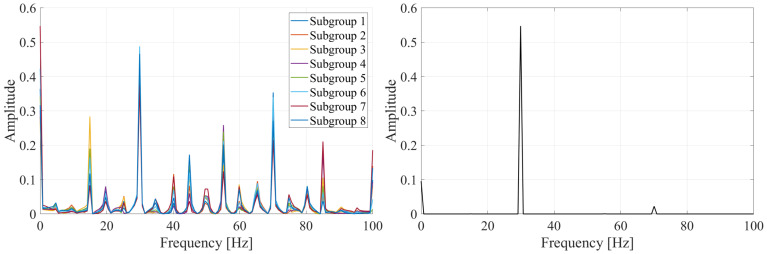
Frequency spectra obtained from the NUFFT analysis of different sensor layouts (**left**) and inter-spectrum of all sensor layouts (**right**) for the oscillation depicted in Figure 13.

To demonstrate the Multi-Sampling process, a simplified harmonic oscillation is analyzed. An undamped harmonic oscillation of EO06, corresponding to a frequency of 30 Hz, is depicted in Figure 13 over two impeller revolutions with period $T_{r}$. Eight sensors are non-uniformly distributed around the impeller circumference, as shown in Figure 13. The deflections measured by these sensors are represented in Figure 13 by colored crosses. Different sensor configurations are generated by removing the data from one sensor, resulting in eight sensor subgroups, each containing data from seven sensors. The frequency spectra obtained from the NUFFT analysis of these sensor layouts are presented in Figure 14. In all sensor layouts, the frequency with the highest amplitude corresponds to the true frequency of the harmonic signal, i.e., 30 Hz. However, due to the under-sampled nature of the data, several replications appear. The frequencies and amplitudes of these replications depend on the specific sensor layout.

To remove these replications, the inter-spectrum of all sensor configurations is calculated through Equation (17). This process effectively suppresses frequencies that exhibit low amplitudes in any of the sensor layouts, as illustrated in the inter-spectrum shown in Figure 14. Nonetheless, if a replication has a considerable amplitude in every sensor configuration, it may not be completely eliminated from the inter-spectrum. In our example, the replication at 70 Hz is not entirely removed from the inter-spectrum. However, its amplitude in the inter-spectrum is significantly smaller than that of the true frequency. This reduction occurs because the inter-spectrum is calculated as the product of the frequency spectra from the different sensor layouts. Consequently, it is sufficient for a replication to have a relatively small amplitude in even one of the sensor layouts for its amplitude in the inter-spectrum to be substantially reduced.

**Figure 15:**
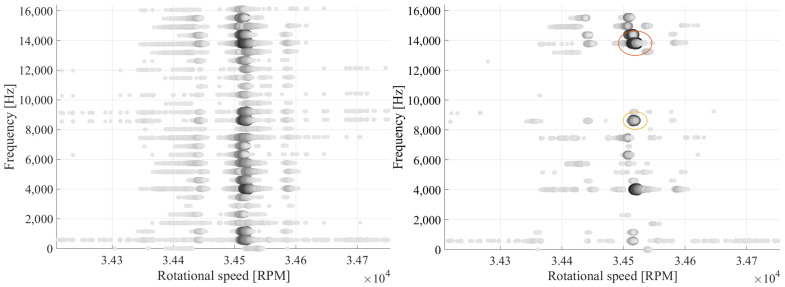
Comparison between the results of the NUFFT (**left**) and the Multi-Sampling (**right**) analysis of the deflection signal illustrated in Figure 11, in which an EO24 vibration occurs.

To illustrate the aliasing effect of the NUFFT analysis as well as the Multi-Sampling method in real experimental data, Figure 15 demonstrates the spectrograms of the blade deflections illustrated in Figure 11. From the deflection signal, a significant vibration can be identified at approximately 34,500 RPM. For the analysis, the deflections are separated into smaller sections, which contain a specific number of revolutions. The spectrum of the sections is plotted over the rotational speed. The rotational speed, where a vibration occurs, can be recognized on the spectrogram of the NUFFT. Nevertheless, an estimation of the vibration frequency cannot be performed through the NUFFT, due to numerous replications that appear. In contrast, the vast majority of the replications are removed with the Multi-Sampling method, while EO24 can be recognized as the frequency with the largest amplitude. Yet, it must be noted that replications, such as EO15 and EO07, can still be observed in the spectrogram of the Multi-Sampling method.

In order to identify the true vibration frequency among the remaining replications, an automated evaluation method based on a statistical approach is employed. Particularly, regions where an amplitude threshold is exceeded are identified in the Multi-Sampling spectrograms. These regions are called Points of Interest (POIs). Since the results of the Multi-Sampling method are heavily influenced by the quality of the deflection signal and the quantity of the BTT sensors, it is possible that a replication is identified as the frequency with the highest amplitude in a resonance crossing. For this reason, multiple frequencies that exceed the amplitude threshold can be identified in a single section. A list of POIs is created for each blade and highlighted in the Multi-Sampling spectrograms with circles.

To prevent a replication from being falsely identified as the vibration frequency, the vibration frequency is determined by considering the POIs over all blades and resonance events in a speed region. Specifically, the weight W of the EOs identified by the POIs is calculated through Equation (18). The weight of an EO is determined by its frequency of occurrence, i.e., how often it is identified by POIs, and by its amplitude, i.e., whether it is the EO with the highest amplitude in a section, through the consideration of weight factor α. By considering the POIs of all blades in a speed region, the vibration frequencies can be reliably identified.

(18) $$W_{EO}=\sum_{b=1}^{B}\sum_{POI_{EO}=1}^{C}\alpha_{b,POI_{EO}}$$

**Figure 16:**
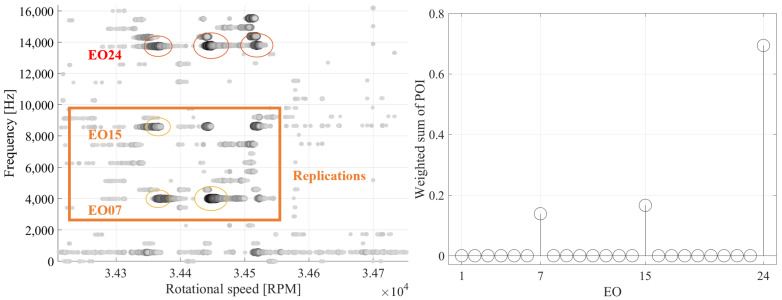
Multi-Sampling method results of a blade (**left**) and weighted sum of POIs (**right**) over all resonance occurrences and blades for an exemplary speed range, in which an EO24 vibration occurs.

The process is depicted in Figure 16, where the results of the Multi-Sampling method of an exemplary blade are presented, along with the weight of the EOs identified by POIs over all blades for a speed region in which an EO24 vibration occurs. The POI with the largest amplitude in a section is highlighted with a red circle, while all others are marked with orange circles. The Multi-Sampling method identifies three resonance occurrences. The true vibration frequency is correctly identified in all of these areas. However, replications that exceed the amplitude threshold are also detected. By considering the results of the Multi-Sampling method over all resonance crossings and blades, the true EO is correctly recognized as the most likely occurring EO. By providing the detected EO to the CFF method, an automated evaluation procedure is enabled, while the number of EOs that are evaluated by the CFF method is greatly reduced.

**Figure 17:**
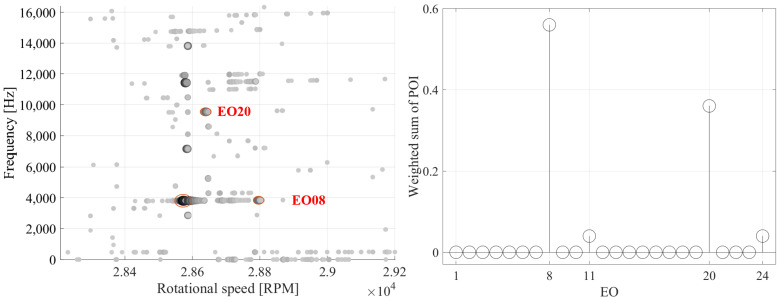
Multi-Sampling method results for a blade (**left**) and weighted sum of POIs (**right**) over all resonance occurrences and blades for an exemplary speed range, in which two vibrations occur.

The Multi-Sampling method enables the possibility of blind analysis of BTT measurements, i.e., prior knowledge of the vibration frequency of the blades is not required. Furthermore, the method can identify more than one vibration frequency. Figure 17 demonstrates the results of a speed region in which two vibrations occur, an EO08 and an E020. Both vibrations are successfully identified.

### 4.5. Estimation of Vibration Properties

For the determination of the vibration properties, a conventional CFF analysis is used. Specifically, the frequencies determined by the Multi-Sampling method are provided as input for a CFF analysis. As a result, a user-defined frequency is not required, and an automated evaluation is enabled.

CFF is a statistical method, which tries to fit sine oscillations to the BTT deflection signals through the LSQM. According to Equation (19), the measured tangential deflection *d* of a blade at a BTT sensor equals an undamped sine oscillation, where *A* expresses the amplitude, ω the angular frequency, *t* the time, and φ the phase of the vibration. *D* expresses the static deflection of the blade, which is caused by the centrifugal forces, untwist, etc.

(19) d(t)=Asin(ωt+φ)+D

As the frequency $f_{b}$ of synchronous blade vibrations is an integer multiple of the rotational frequency $f_{R}$, the following relation can be expressed for the angular frequency (Equation (20)):

(20) $$\omega =2\pi f_{b}=2\pi EOf_{R}$$

According to Equation (21), under the assumption of constant rotational speed during a revolution, a relation can be extracted between the circumferential position in stationary frame θ and the angular frequency:

(21) $$\omega \cdot t=EO\cdot 2\pi f_{R}t=EO\cdot \theta$$

As a result, the tangential deflection of a blade at a BTT sensor can be expressed through Equation (22):

(22) $$\begin{array}{cc} d(\theta ) & =A\sin(EO\theta +\varphi )+D\\ \; & =A_{1}\sin\left(EO\theta \right)+B_{1}\cos\left(EO\theta \right)+D \end{array}$$

As the static component is removed in the deflection calculation method, the static deflection can be neglected. Furthermore, the deflection difference of a blade between two BTT sensors is expressed through Equation (23):

(23) $$\begin{array}{cc} d_{1}-d_{2}=A_{1} & \left(\sin\left(EO\theta_{1}\right)-\sin\left(EO\theta_{2}\right)\right)\\ \; & +B_{1}\left(\cos\left(EO\theta_{1}\right)-\cos\left(EO\theta_{2}\right)\right) \end{array}$$

By using the deflection differences of each sensor combination in a revolution, a linear equation system is formed according to Equation (24). Vector $\bar{d}$ symbolizes the deflection differences determined from the TOA data, *M* is the coefficient matrix, while $\bar{y}$ contains the unknowns to be determined.

(24) $$\begin{array}{c} \left(\begin{array}{c} d_{1}-d_{2}\\ d_{1}-d_{3}\\ \vdots \\ d_{S-1}-d_{S} \end{array}\right)=\left(\begin{array}{cc} \sin\left(EO\theta_{1}\right)-\sin\left(EO\theta_{2}\right) & \cos\left(EO\theta_{1}\right)-\cos\left(EO\theta_{2}\right)\\ \sin\left(EO\theta_{1}\right)-\sin\left(EO\theta_{3}\right) & \cos\left(EO\theta_{1}\right)-\cos\left(EO\theta_{3}\right)\\ \vdots & \vdots \\ \sin\left(EO\theta_{S-1}\right)-\sin\left(EO\theta_{S}\right) & \cos\left(EO\theta_{S-1}\right)-\cos\left(EO\theta_{S}\right) \end{array}\right)\left(\begin{array}{c} A_{1}\\ B_{1} \end{array}\right) \end{array}$$

(25) $$\begin{array}{c} \;\bar{d}=M\cdot \bar{y} \end{array}$$

A key advantage for employing the deflection differences is the extension of vector $\bar{d}$ and, thus, of the input data of the LSQM. As a result, the method is less affected by noise [68]. Moreover, the elimination of the static deflection leads to a reduction in the parameters to be determined. Hence, the stability and accuracy of the method are further enhanced.

The linear equation system is subsequently solved for each blade through the LSQM. Specifically, parameters $A_{1}$ and $B_{1}$ are obtained by minimizing the Squared Sum of Differences *J*, which is defined through Equation (26) [69].

(26) $$J={\left(\bar{d}-M\cdot \bar{y}\right)}^{T}\left(\bar{d}-M\cdot \bar{y}\right)$$

To minimize the Squared Sum of Differences, the gradient of *J* in respect to $\bar{y}$, as defined in Equation (27), must be equal to zero.

(27) $$\frac{\partial J}{\partial \bar{y}}=-2M^{T}\bar{d}+2M^{T}M\bar{y}\overset{!}{=}0$$

The parameter vector can be then determined through Equation (28).

(28) $$\bar{y}={\left(M^{T}M\right)}^{-1}\left(M^{T}\bar{d}\right)$$

To evaluate the quality of the fit between the sine function and the deflection data, the residuals of the operation can be used. These are defined through Equation (29). From the residuals, a single error term can be derived using different approaches, such as the Euclidean norm or the mean absolute error [70].

(29) $$\bar{r}=\bar{d}-M\cdot \bar{y}$$

Finally, the amplitude and phase of the vibration can be determined using parameters $A_{1}$ and $B_{1}$ through the following relations (Equation (30)):

(30) $$A=\sqrt{A_{1}^{2}+B_{1}^{2}}\;,\;\varphi =\tan^{-1}\left(\frac{A_{1}}{B_{1}}\right)$$

**Figure 18:**
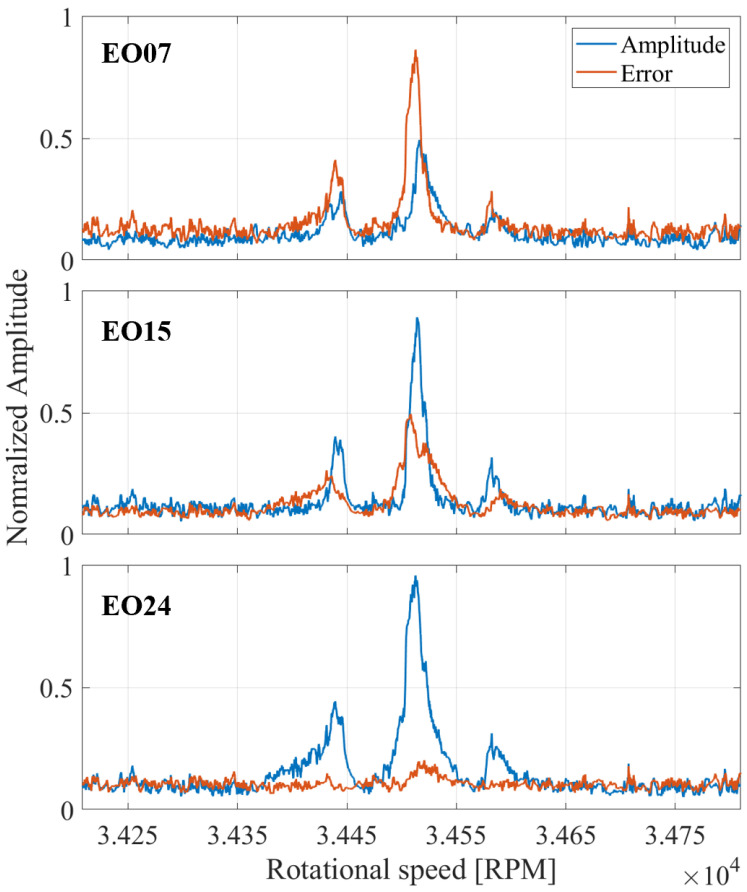
Illustration of the CFF results when applying the frequencies detected by the Multi-Sampling method on the deflection signal depicted in Figure 11.

Figure 18 illustrates the results of the CFF method when applying the frequencies detected by the Multi-Sampling method on the deflection signal depicted in Figure 11. The amplitude and the error term are presented against the rotational speed for EO07, EO15, and EO24. By evaluating the error term of the examined EOs, the true vibration frequency can be determined. EO24 can be identified as the true vibration frequency due to its exceptionally low error term, which indicates a good fit with the deflection signal. In contrast, both EO07 and EO15 have significantly higher error terms, which are comparable to the vibration amplitude. Hence, the CFF method confirms the results of the Multi-Sampling method, which correctly detected EO24 as the most likely occurring EO.

**Figure 19:**
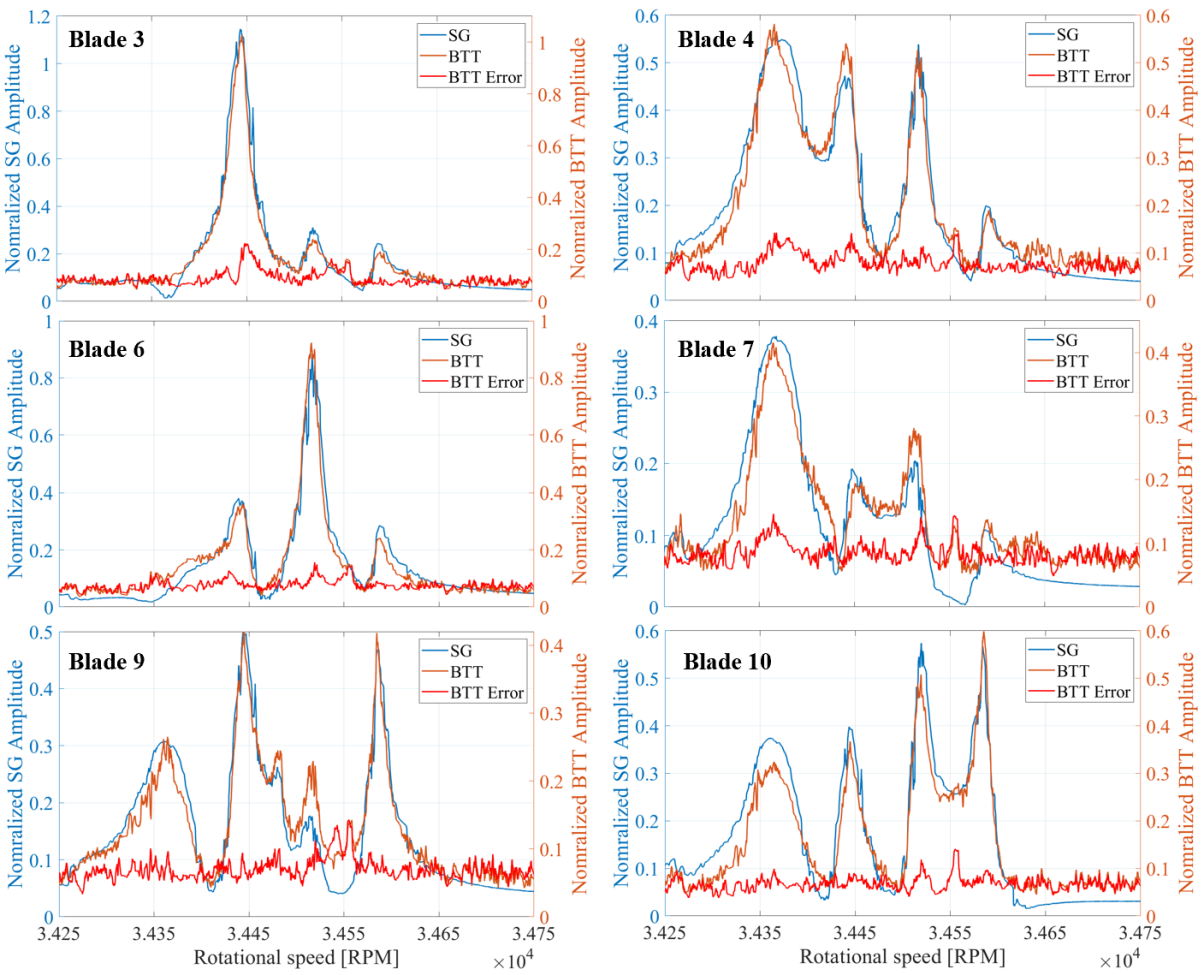
Amplitude comparison of an EO24 vibration between SG and BTT for six exemplary blades.

**Figure 20:**
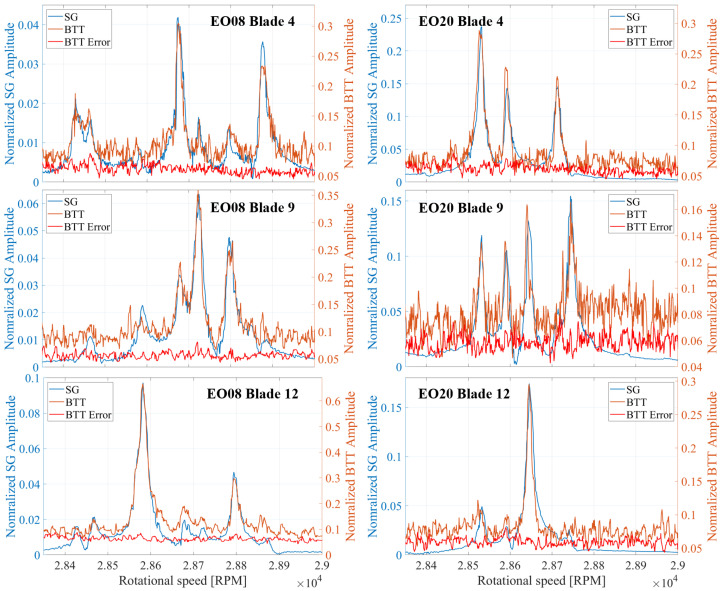
Amplitude comparison of two vibrations (EO08 and EO20) occurring in the same speed region between SG and BTT for three exemplary blades.

**Figure 21:**
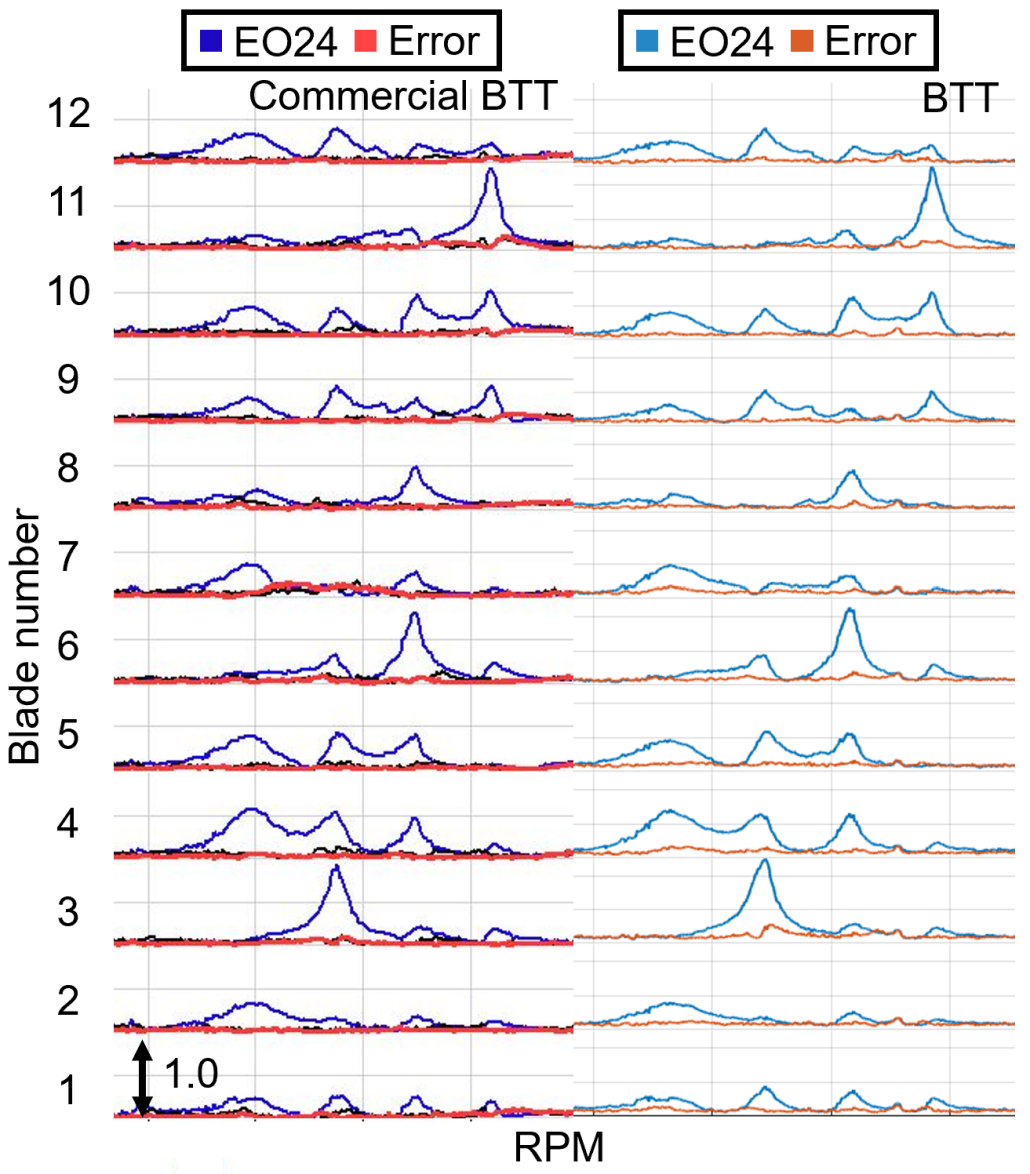
Comparison of the CFF results of an EO24 vibration between a commercial BTT system and the evaluation procedure demonstrated in this paper.

**Figure 22:**
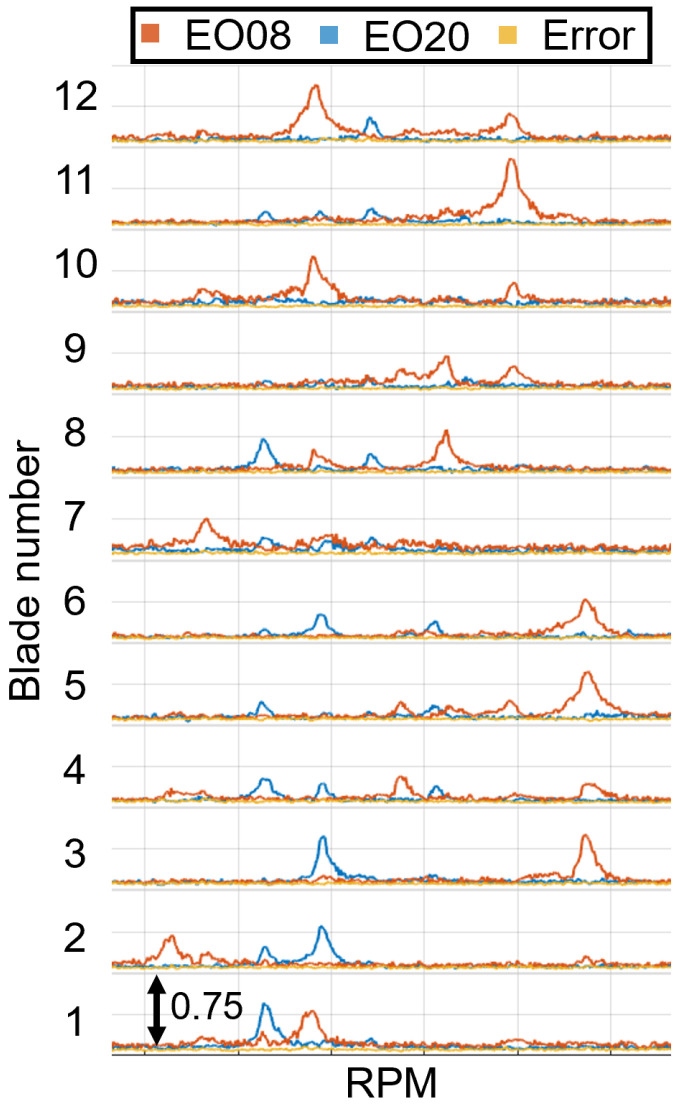
Stack plot diagram of the amplitude versus speed curve for two vibrations (EO08 and EO20) occurring in the same speed region.

The vibration properties determined with the CFF analysis are compared with those obtained from SG measurements. The experimental data were acquired from a turbocharger test bench under real operating conditions. A detailed description of the test bench and a comprehensive analysis of the vibration measurements can be found in previous publications [11,57]. The amplitude versus speed curve for some blades is presented for both measurement systems in Figure 19 for a speed range where an EO24 vibration occurs, and in Figure 20 for a speed region where two vibrations, an EO20 and an EO08, appear. Stack plot diagrams showing the amplitude versus speed curve of all blades, as determined by the proposed evaluation procedure, are presented for both speed regions in Figure 21 and Figure 22 respectively. The vibration properties of the SG signal are determined through an STFT analysis. The comparison demonstrates a direct correlation between the SG measurements and the evaluated BTT data. Remarkably, a similar amplitude curve is observed. Here, it should be mentioned that, since SGs measure strain and BTT measures tangential blade deflection, a direct comparison between the vibration magnitudes is not possible. Hence, the validation is restricted in the qualitative comparison of the amplitude.

**Figure 23:**
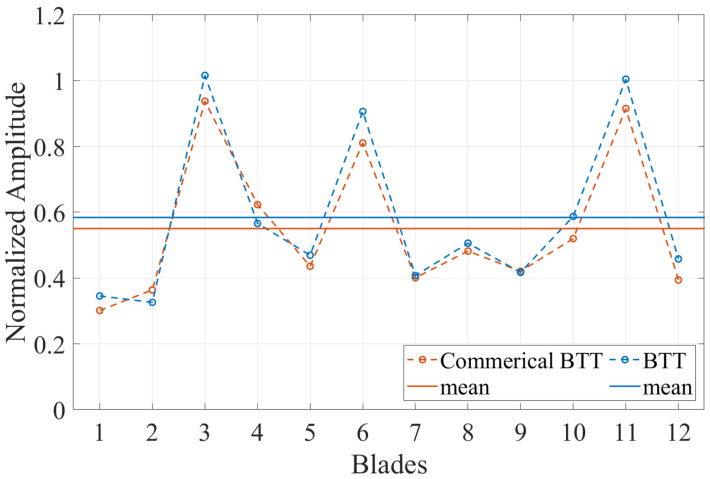
Amplitude comparison of an EO24 vibration between a commercial BTT system and the evaluation procedure demonstrated in this paper.

The proposed evaluation procedure is further validated by performing a comparison with a commercial BTT system, which receives the same raw electrical signal and determines the blade deflections using an OPR sensor. For this comparison, a BTT system of the company Hood Technology is used. The amplitude is depicted over the speed, along with the error term in a stack plot diagram in Figure 21 for an exemplary speed range in which an EO24 vibration occurs. An identical amplitude curve is observed. Moreover, a comparison between the vibration magnitudes is provided in Figure 23, where the amplitude is presented for all blades. The proposed evaluation procedure yields slightly higher amplitude values of approximately 5%. Several factors throughout the evaluation process can contribute to these minor amplitude differences. These factors involve variations in the determination of the TOAs and differences in the deflection calculation method, including the computation of the static component and noise reduction, as well as deviations in the CFF method.

## 5. Conclusions and Outlook

This paper presents a comprehensive evaluation procedure for BTT data, encompassing the entire process from determining TOAs from raw signals to calculating blade vibration properties. The proposed methods aim to address significant challenges associated with BTT, namely the reliance on the OPR sensor for calculating blade deflections, and the problematic estimation of vibration frequencies due to under-sampled deflection signals. The main contributions of this work are as follows: (1) the introduction of the novel Rotor Position method for accurate OPR-free calculation of blade deflections, and (2) the development of an improved Multi-Sampling method for reliable vibration frequency assessment.

First, the TOAs are allocated to the blades by utilizing blade “fingerprints” derived from manufacturing tolerances. Specifically, the angle between the blades is used for blade recognition. The method is successfully applied to two impellers, demonstrating that consistent blade identification can be performed regardless of the manufacturing process.

Subsequently, the blade deflections are calculated through the Rotor Position method. Unlike other OPR-free methods that generate a reference time, which is then subtracted from the measured TOAs for the calculation of the blade deflections, the proposed method calculates the blade deflections in the angle domain by subtracting the sensor positions from the blade circumferential positions. The blade circumferential positions are obtained by integrating the rotor rotational frequency over time while accounting for the actual blade positions on the impeller. By considering all TOAs within a revolution, a highly sampled rotational frequency is derived that effectively captures speed variations occurring within a revolution. To minimize the influence of blade vibrations on the rotational frequency, the rotational frequency of the rotor is obtained by averaging the rotational frequencies of all BTT sensors. The dynamic blade deflections are obtained by subtracting the static component, while the noise of the deflection signal is reduced by employing time-synchronous averaging techniques.

By completely eliminating the need for an OPR sensor, the accuracy and applicability of BTT are enhanced. Specifically, a more precise estimation of the blade deflections is enabled through an OPR-free calculation procedure, as the blade deflections are no longer affected by shaft vibrations, while speed variations within a revolution can be accounted for. Moreover, the implementation possibilities of BTT are enhanced, allowing its application in systems where an OPR sensor cannot be instrumented due to space constraints.

The blade deflections are used by the CFF method for the calculation of the vibration amplitude and phase. The Rotor Position method is validated by comparison with SG measurements using experimental data acquired under real operating conditions from a turbocharger test bench [11,57]. The comparison demonstrates a direct correlation between the SG measurements and the evaluated BTT data. In addition, a comparison with a commercial BTT system is performed. Similarly, an identical amplitude evolution is observed for all blades, which exhibit the same vibration magnitudes in both evaluations.

In future works, the accuracy of the proposed BTT evaluation process could be further improved by employing an advanced CFF method capable of adjusting sensor positions by considering speed variations within a revolution, as well as the blade deflections at these sensors. A CFF method that accounts for speed variation within a revolution was proposed by Fan C. et al. [19]. In this method, a relation between a reference time and its corresponding angle is established through polynomial fitting. The speed change is then obtained by taking the first derivative of the angle with respect to time. Subsequently, this speed change is used to calculate the sensor positions for each blade. In addition, an alternative CFF method that considers the impact of blade deflections on the sensor positions was introduced by Liu et al. [22]. In this approach, the sensor positions are adjusted for each blade and revolution according to the occurring deflections. By combining these two methods, an improved CFF method could be derived, further enhancing the accuracy of BTT.

To overcome challenges associated with under-sampled deflection signals, the detection of the vibration frequency is performed via an improved Multi-Sampling method. This method utilizes the distinctive aliasing patterns generated by different non-equidistant sensor layouts to identify the vibration frequency. Initially, the frequency spectra of the different sensor layouts are calculated through NUFFT analysis. Subsequently, to isolate the vibration frequency common to all sensor layouts, the inter-spectrum is calculated. By employing an automated analysis of the inter-spectrum across all blades and resonance events within a given speed range, the vibration frequency is identified. This approach enables the blind analysis of BTT measurements and can identify multiple vibration frequencies. Moreover, it demonstrates robustness across variable speed conditions and does not require strict sensor layouts. The proposed method enhances the capabilities of BTT by providing reliable assessment of vibration frequencies, thus eliminating the need for parallel strain gauge measurements for frequency identification.

Despite these advantages, vibrations with small amplitudes may still not be detected, and the results are heavily influenced by the quality of the deflection signal and the number of BTT sensors. Although this method does not require strict sensor layouts, it would be beneficial to investigate whether an optimal sensor configuration can be mathematically determined to ensure the reliable identification of multiple frequency components across a wide frequency spectrum. Additionally, the results of this method are significantly affected by various parameters related to the NUFFT analysis, such as the resolution and range of the frequency domain, the number of revolutions included in each analysis section, and the choice of windowing and overlapping functions. Future research should concentrate on optimizing these parameters in relation to the sensor configuration and the vibration characteristics of the investigated impeller. Furthermore, while this method cannot currently provide accurate estimations of the vibration amplitude, refining the NUFFT analysis could improve the assessment of vibration magnitudes.

Finally, as an alternative to the CFF method, the Multi-Sampling method can be used in combination with signal reconstruction techniques proposed in previous publications [45,46,50,51] for calculating vibration properties. In this context, the Multi-Sampling method can be employed to identify the frequency components of the under-sampled BTT signal, which can then serve as inputs for the signal reconstruction methods.

## Data Availability

Data are contained within the article.

## Nomenclature

*b*	Blade [-]
*c*	Blade located between first blade and observed blade *b* [-]
*d*	Blade deflection [m]
$d_{d}$	Dynamic blade deflection [m]
$d_{d,sm}$	Smoothed dynamic blade deflection [m]
$d_{st}$	Static component of blade deflection [m]
$\bar{d}$	Deflection difference vector [m]
*f*	Frequency [Hz]
$f_{b}$	Blade vibration frequency [Hz]
$f_{R}$	Rotational frequency of rotor [Hz]
$f_{s}$	Rotational frequency calculated at sensor *s* [Hz]
*i*	Revolution [-]
*k*	Sensor subgroup [-]
*p*	Non-uniformly distributed sample point [-]
*q*	Query point of rotor time and rotational frequency [-]
*r*	Impeller radius at axial plane of BTT [m]
$\bar{r}$	Residuals vector [-]
*s*	Sensor [-]
*t*	Time [s]
$t_{R}$	Rotor time [s]
*x*	Value at sample point p [-]
$\bar{y}$	Parameter vector [m]
*A*	Amplitude [m]
$A_{1}$	Unknown parameter [m]
*B*	Number of blades [-]
$B_{1}$	Unknown parameter [m]
*C*	Number of POIs of an EO in a blade [-]
*D*	Static deflection [m]
*I*	Number of revolutions [-]
*J*	Squared Sum of Differences [-]
*K*	Number of sensor subgroups [-]
*L*	Smoothing window [-]
*M*	Coefficient matrix [-]
*N*	Number of non-uniformly distributed sample points *p* [-]
*P*	Spectrum [-]
$P_{IS}$	Inter-spectrum [-]
$P_{k}$	Spectrum of sensor configuration *k* [-]
*Q*	Number of query points [-]
*S*	Number of sensors [-]
$T_{r}$	Revolution period of the impeller [s]
$W_{EO}$	Weight of EO [-]
α	Weight factor of a POI [-]
θ	Circumferential position in stationary frame [rad]
$\theta_{s}$	Circumferential position of sensor *s* [rad]
ϕ	Blade circumferential position [rad]
$\phi_{1\to b}$	Angle between blades 1 and *b* [rad]
$\phi_{b\to b+1}$	Angle between two consecutive blades [rad]
$\phi_{R}$	Rotor circumferential position [rad]
φ	Phase of vibration [rad]
ω	Angular frequency [rad/s]
Δϕ	Deviation of blade spacing from targeted value [rad]
BTT	Blade Tip Timing
CFF	Circumferential Fourier Fit
CS	Compressive Sensing
DAU	Data Acquisition Unit
EO	Engine Order
FEM	Finite Element Method
FFT	Fast Fourier Transform
LSQM	Least Square Method
MPR	Multiple-per-Revolution
MUSIC	Multiple Signal Classification
NUFFT	Non-Uniform Fast Fourier Transform
OPR	Once-per-Revolution
POI	Point of Interest
RPM	Revolutions per Minute [1/min]
SG	Strain Gauges
SGF	Savitzky–Golay filter
SLF	Straight Line Fitting
STFT	Short-Time Fourier Transform
TOA	Time of Arrival

## References

[1] Kemp N.H., Sears W.R. (1953). Aerodynamic Interference Between Moving Blade Rows. J. Aeronaut. Sci..

[2] Kemp N.H., Sears W.R. (1955). The Unsteady Forces Due to Viscous Wakes in Turbomachines. J. Aeronaut. Sci..

[3] Müller T.R., Waldherr C., Kovachev N., Rakut C., Esper A., Lenzen C., Wunderlich M. (2018). Blade Forces. Final Report, FVV Project no.1189.

[4] Chung M.H., Wo A.M. (1997). Navier–Stokes and Potential Calculations of Axial Spacing Effect on Vortical and Potential Disturbances and Gust Response in an Axial Compressor. J. Turbomach..

[5] Kreuz-Ihli T. (2001). Strömungsinduzierte Schaufelschwingungen in Leitgitterlosen Radialturbinen. Ph.D. Thesis.

[6] Klaus M. (2007). Strömungsinduzierte Schaufelschwingungen in Radialturbinen mit beschaufeltem Spiralgehäuse. Ph.D. Thesis.

[7] Netzhammer S., Vogt D.M., Kraetschmer S., Leweux J., Koengeter A. (2017). Aerodynamic Excitation Analysis of Radial Turbine Blades Due to Unsteady Flow From Vaneless Turbine Housings. Turbo Expo: Power for Land, Sea, and Air, 7B: Structures and Dynamics.

[8] Beirow B., Kühhorn A., Nipkau J. (2009). On the Influence of Strain Gauge Instrumentation on Blade Vibrations of Integral Blisk Compressor Rotors Applying a Discrete Model. Turbo Expo: Power for Land, Sea, and Air, 6: Structures and Dynamics, Parts A and B.

[9] Giersch T., Hönisch P., Beirow B., Kühhorn A. (2013). Forced Response Analyses of Mistuned Radial Inflow Turbines. J. Turbomach..

[10] Klauke T. (2007). Schaufelschwingungen Realer Integraler Verdichterräder im Hinblick auf Verstimmung und Lokalisierung. Ph.D. Thesis.

[11] Sasakaros M., Schafferus M., Wirsum M., Zobel A., Vogt D., Nakos A., Beirow B. (2024). Experimental Investigation of Synchronous-Flow-Induced Blade Vibrations on a Radial Turbine. Int. J. Turbomachinery, Propuls. Power.

[12] Zielinski M., Ziller G. (2005). Noncontact Blade Vibration Measurement System for Aero Engine Application. Meas. Sci. Technol..

[13] Schlagwein G., Schaber U. (2006). Non-Contact Blade Vibration Measurement Analysis Using a Multi-Degree-of-Freedom Model. Proc. Inst. Mech. Eng. Part A J. Power Energy.

[14] Stephan C., Berthillier M., Joseph L., Talon A. (2008). Tip-Timing Data Analysis for Mistuned Bladed Discs Assemblies. Turbo Expo: Power for Land, Sea, and Air, Volume 5: Structures and Dynamics, Parts A and B.

[15] Dimitriadis G., Carrington I.B., Wright J.R., Cooper J.E. (2002). Blade-Tip Timing Measurement of Synchronous Vibrations of Rotating Bladed Assemblies. Mech. Syst. Signal Process..

[16] Carrington I.B., Wright J.R., Cooper J.E., Dimitriadis G. (2001). A Comparison of Blade Tip Timing Data Analysis Methods. Proc. Inst. Mech. Eng. Part G J. Aerosp. Eng..

[17] Zhang J., Duan F., Jiang J. Analysis of a Signal Preprocessing Method for Blade Tip-Timing without the Once-per Revolution Sensor. Proceedings of the 2017 2nd International Conference on Electrical, Automation and Mechanical Engineering (EAME 2017).

[18] Harvey A.F., Cerna M. (1993). The Fundamentals of FFT-Based Signal Analysis and Measurement in LabVIEW and LabWindows.

[19] Fan C., Wu Y., Russhard P., Wang A. (2020). An Improved Blade Tip-timing Method for Vibration Measurement of Rotating Blades During Transient Operating Conditions. J. Vib. Eng. Technol..

[20] Przysowa R. (2015). The Analysis Of Synchronous Blade Vibration Using Linear Sine Fitting. J. KONBiN.

[21] Kaźmierczak K., Przysowa R. (2015). Standard Sine Fitting Algorithms Applied To Blade Tip Timing Data. J. KONBiN.

[22] Zhibo L., Fajie D., Niu G., Ma L., Jiajia J., Fu X. (2020). An Improved Circumferential Fourier Fit (CFF) Method for Blade Tip Timing Measurements. Appl. Sci..

[23] Jousselin O. (2013). Development of Blade Tip Timing Techniques in Turbomachinery. Ph.D. Thesis.

[24] Beauseroy P., Lengellé R. (2007). Nonintrusive turbomachine blade vibration measurement system. Mech. Syst. Signal Process..

[25] Vercoutter A., Lardies J., Berthillier M., Talon A., Burgardt B. (2013). Improvement of Compressor Blade Vibrations Spectral Analysis From Tip Timing Data: Aliasing Reduction. Turbo Expo: Power for Land, Sea, and Air, Volume 7A: Structures and Dynamics.

[26] Kharyton V., Dimitriadis G., Defise C. (2017). A Discussion on the Advancement of Blade Tip Timing Data Processing. Turbo Expo: Power for Land, Sea, and Air, Volume 7B: Structures and Dynamics.

[27] Sasakaros M., Mann L., Schafferus M., Wirsum M. (2024). Determination of Vibration Properties and Reliable Frequency Estimation for Synchronous Vibrations Through Improved Blade-Tip-Timing Techniques Without a Once-per-Revolution Sensor. Volume 10B: Structures and Dynamics—Fatigue, Fracture, and Life Prediction; Probabilistic Methods; Rotordynamics; Structural Mechanics and Vibration.

[28] Russhard P. Derived once per rev signal generation for Blade Tip Timing systems. Proceedings of the IET & ISA 60th International Instrumentation Symposium 2014.

[29] Chen K., Wang W., Zhang X., Zhang Y. (2019). New step to improve the accuracy of blade tip timing method without once per revolution. Mech. Syst. Signal Process..

[30] He C., Antoni J., Daga A.P., Li H., Chu N., Lu S., Li Z. (2021). An Improved Key-Phase-Free Blade Tip-Timing Technique for Nonstationary Test Conditions and Its Application on Large-Scale Centrifugal Compressor Blades. IEEE Trans. Instrum. Meas..

[31] Daga A.P., Garibaldi L., He C., Antoni J. (2021). Key-Phase-Free Blade Tip-Timing for Nonstationary Test Conditions: An Improved Algorithm for the Vibration Monitoring of a SAFRAN Turbomachine from the Surveillance 9 International Conference Contest. Machines.

[32] Wang W., Zhang X., Hu D., Zhang D., Allaire P. (2020). A novel none once per revolution blade tip timing based blade vibration parameters identification method. Chin. J. Aeronaut..

[33] Zhang J., Zhang L., Ding K., Duan L. (2018). Blade Tip-timing Technology with Multiple Reference Phases for Online Monitoring of High-speed Blades under Variable-speed Operation. Meas. Sci. Rev..

[34] Fan C., Wu Y., Russhard P., Ruan C., Wang A. (2021). A blade tip-timing method without once-per-revolution sensor for blade vibration measurement in gas turbine engines. Trans. Can. Soc. Mech. Eng..

[35] Fan Z., Li H., Dong J., Zhao X. (2022). An improved multiple per revolution-based blade tip timing method and its applications on large-scale compressor blades. Mech. Syst. Signal Process..

[36] Wang W., Chen K., Zhang X., Li W. (2022). A novel method to improve the precision of BTT under rapid speed fluctuation conditions. Mech. Syst. Signal Process..

[37] Guo H., Duan F., Zhang J. (2016). Blade resonance parameter identification based on tip-timing method without the once-per revolution sensor. Mech. Syst. Signal Process..

[38] Wang Z., Yang Z., Wu S., Li H., Tian S., Zhang X., Yan R., Chen X. (2021). An OPR-Free Blade Tip Timing Method for Rotating Blade Condition Monitoring. IEEE Trans. Instrum. Meas..

[39] Lin J., Hu Z., Chen Z., Yang Y., Xu H. (2016). Sparse reconstruction of blade tip-timing signals for multi-mode blade vibration monitoring. Mech. Syst. Signal Process..

[40] Pan M., Yang Y., Guan F., Hu H., Xu H. (2017). Sparse Representation Based Frequency Detection and Uncertainty Reduction in Blade Tip Timing Measurement for Multi-Mode Blade Vibration Monitoring. Sensors.

[41] Pan M., Guan F., Hu H., Yang Y., Xu H. Compressed sensing based on dictionary learning for reconstructing blade tip timing signals. Proceedings of the 2017 Prognostics and System Health Management Conference (PHM-Harbin).

[42] Chen Z., Sheng H., Xia Y. (2021). Multi-coset angular sampling-based compressed sensing of blade tip-timing vibration signals under variable speeds. Chin. J. Aeronaut..

[43] Bouchain A., Picheral J., Lahalle E., Chardon G., Vercoutter A., Talon A. (2019). Blade vibration study by spectral analysis of tip-timing signals with OMP algorithm. Mech. Syst. Signal Process..

[44] Xu J., Qiao B., Liu J., Ao C., Teng G., Chen X. (2021). Sparse reconstruction for blade tip timing signal using generalized minimax-concave penalty. Mech. Syst. Signal Process..

[45] Dong J., Li H., Fan Z., Zhao X., Wei D., Chen Y. (2023). Characteristics analysis of blade tip timing signals in synchronous resonance and frequency recovery based on subspace pursuit algorithm. Mech. Syst. Signal Process..

[46] Dong J., Li H., Cao H., Fan Z., Chen Y. (2023). An improved blade tip timing dual-probe method of synchro-resonance frequency identification for blade damage detection. Mech. Syst. Signal Process..

[47] Wu S., Russhard P., Yan R., Tian S., Wang S., Zhao Z., Chen X. (2020). An Adaptive Online Blade Health Monitoring Method: From Raw Data to Parameters Identification. IEEE Trans. Instrum. Meas..

[48] Chen S., Yang Y., Hu H., Guan F., Shen G., Bian Z., Guo H. (2021). Interpolation method for wideband signal reconstruction based on blade tip timing measurement. Measurement.

[49] Chen S., Yang Y., Hu H., Guan F., Shen G., Bian Z., Guo H. (2022). Blind interpolation for multi-frequency blade tip timing signals. Mech. Syst. Signal Process..

[50] Cao J., Yang Z., Teng G., Chen X. (2023). Coprime and nested samplings-based spectrum reconstruction in blade tip timing. Mech. Syst. Signal Process..

[51] Cao J., Yang Z., Tian S., Teng G., Chen X. (2023). Active aliasing technique and risk versus error mechanism in blade tip timing. Mech. Syst. Signal Process..

[52] Wang Z., Yang Z., Wu S., Li H., Tian S., Chen X. (2020). An Improved Multiple Signal Classification for Nonuniform Sampling in Blade Tip Timing. IEEE Trans. Instrum. Meas..

[53] Liu Z., Duan F., Niu G., Ye D., Feng J., Cheng Z., Fu X., Jiang J., Zhu J., Liu M. (2022). Reconstruction of blade tip-timing signals based on the MUSIC algorithm. Mech. Syst. Signal Process..

[54] Wang Z., Yang Z., Li H., Wu S., Tian S., Chen X. (2021). Robust sparse representation model for blade tip timing. J. Sound Vib..

[55] Wang Z., Yang Z., Li H., Cao J., Tian S., Chen X. (2022). Automatic tracking of natural frequency in the time–frequency domain for blade tip timing. J. Sound Vib..

[56] Wang Z., Yang Z., Teng G., Yan R., Tian S., Li H., Cao J., Chen X. (2023). Amplitude-Identifiable MUSIC (Aid-MUSIC) for Asynchronous Frequency in Blade Tip Timing. IEEE Trans. Ind. Inform..

[57] Esper A., Lenzen C., Wirsum M. Commissioning of a Test Stand for Turbocharger Investigations at Constant Turbine Inlet Temperatures. Proceedings of the 17th International Symposium on Transport Phenomena and Dynamics of Rotating Machinery (ISROMAC2017).

[58] Greengard L., Lee J. (2004). Accelerating the Nonuniform Fast Fourier Transform. SIAM Rev..

[59] Duijndam A.J.W., Schonewille M.A. (1999). Nonuniform fast Fourier transform. Geophysics.

[60] Beylkin G. (1995). On the Fast Fourier Transform of Functions with Singularities. Appl. Comput. Harmon. Anal..

[61] Beylkin G. (1998). On Applications of Unequally Spaced Fast Fourier Transforms. Math. Geophys. Summer Sch. Stanf..

[62] Potts D., Steidl G., Tasche M. (2001). Fast Fourier Transforms for Nonequispaced Data: A Tutorial. Modern Sampling Theory: Mathematics and Applications.

[63] Potts D., Steidl G. (2003). Fast Summation at Nonequispaced Knots by NFFT. Siam J. Sci. Comput..

[64] Fessler J., Sutton B. (2003). Nonuniform Fast Fourier Transforms Using Min-Max Interpolation. IEEE Trans. Signal Process..

[65] Ruiz-Antolín D., Townsend A. (2018). A Nonuniform Fast Fourier Transform Based on Low Rank Approximation. SIAM J. Sci. Comput..

[66] Dutt A., Rokhlin V. (1993). Fast Fourier Transforms for Nonequispaced Data. SIAM J. Sci. Comput..

[67] Potter S.F., Gumerov N.A., Duraiswami R. Fast interpolation of bandlimited functions. Proceedings of the 2017 IEEE International Conference on Acoustics, Speech and Signal Processing (ICASSP).

[68] Montgomery D., Peck E., Vining G. (2021). Introduction to Linear Regression Analysis.

[69] Kay S.M. (1993). Fundamentals of Statistical Signal Processing: Estimation Theory.

[70] Willmott C.J., Matsuura K. (2005). Advantages of the mean absolute error (MAE) over the root mean square error (RMSE) in assessing average model performance. Clim. Res..

